# Natural Inhibitors of Cholinesterases: Chemistry, Structure–Activity and Methods of Their Analysis

**DOI:** 10.3390/ijms24032722

**Published:** 2023-02-01

**Authors:** Natalia Smyrska-Wieleba, Tomasz Mroczek

**Affiliations:** Department of Chemistry of Natural Products, Medical University of Lublin, 1 Chodzki Str., 20-093 Lublin, Poland

**Keywords:** natural products, acetylcholinesterase inhibitors, butyrylcholinesterase inhibitors, Alzheimer’s disease, central nervous system

## Abstract

This article aims to provide an updated description and comparison of the data currently available in the literature (from the last 15 years) on the studied natural inhibitors of cholinesterases (IChEs), namely, acetylcholinesterase (AChE) and butyrylcholinesterase (BuChE). These data also apply to the likely impact of the structures of the compounds on the therapeutic effects of available and potential cholinesterase inhibitors. IChEs are hitherto known compounds with various structures, activities and origins. Additionally, multiple different methods of analysis are used to determine the cholinesterase inhibitor potency. This summary indicates that natural sources are still suitable for the discovery of new compounds with prominent pharmacological activity. It also emphasizes that further studies are needed regarding the mechanisms of action or the structure–activity correlation to discuss the issue of cholinesterase inhibitors and their medical application.

## 1. Introduction

Cholinesterase inhibitors are chemical compounds that impair the activity of cholinesterases: AChE and BuChE. They reduce the hydrolysis of the neurotransmitters acetylcholine (ACh) (acetylcholinesterase inhibitors) and butyrylcholine (butyrylcholinesterase inhibitors), thereby increasing their levels in the body (brain, blood and nerve tissue). Naturally occurring cholinesterase inhibitors affect esterases in a reversible manner [1]. 

IChE drugs currently used in medicine are synthetically derived. The majority of them originate from natural substances. One of them, tacrine, was approved for treatment, and it has been used similarly to donepezil, galanthamine (**1**) and rivastigmine. Unfortunately, the first of them causes hepatotoxicity, while the others have side effects including insomnia, diarrhea, nausea and vomiting [2].

IChEs (BuChE and AChE) also show therapeutic activity when applied in treatments for myasthenia gravis, myopathies, disorders associated with peripheral nerve damage, impaired conduction of nervous stimuli, and diseases associated with dementia, such as vascular dementia and Alzheimer’s and Parkinson’s diseases [3,4,5]. 

The mechanisms causing Alzheimer’s disease (AD) are not entirely understood. In patients with AD, marked decreases in neurotransmitter levels in the cells are observed. In particular, the concentration of acetylcholine is reduced, together with dopamine, glutamate, serotonin and norepinephrine [6].

Currently, due to the limited knowledge of effective methods of treating the causes of these disorders, therapies, as before, are mainly based on symptomatic treatments (except for Aduhelm^®^ Aducanumab, which underwent accelerated FDA (U.S. Food and Drug Administration) approval [7]). Studies indicate that an increase in the level of cholinergic transmission in patients with AD (increase in ACh) mitigates disease progression. It also has a beneficial effect on cognitive functions and improves the patient’s mood, despite the reduction in cholinergic neurons in the brain [1,5,6]. This can be accomplished by inhibiting the hydrolytic enzyme that decomposes acetylcholine (AChE) after its release from neurons to the synaptic area. There are some studies that suggest that maintaining acetylcholine prevents the formation of senile plaques through its indirect impact on the activation of α-secretase. This is a result of the activation of protein kinase C upon stimulation by the ACh receptor muscarinic M1 [5].

AChE and BuChE can be distinguished in the central nervous system [8]. Both cholinesterases influence the distribution of ACh.

With the level of advancement of AD, the increased function of BuChE with a decrease in AChE was observed [9]. There are reports suggesting that AChE impacts the progression of dementia diseases by increasing the expression of Aβ amyloid precursor, neuronal apoptosis and the aggregation of AChE-Aβ amyloid, which is more toxic than the protein itself [1].

For the mitigation of symptoms such as a decline in cognition, listlessness and mood swings, inhibitors of BuChE may be helpful due to the presence of the enzyme observed in the structures responsible for these functions in the brain (thalamic nucleus and glia). BuChE was also present in pathological structures: senile plaques and neurofibrillary tangles in patients with AD [3,8]. During tests in normal mice, these stimulants showed an increasing tendency of ACh release in the brain. These functions are controlled by the areas of the forebrain. This can lead to the conclusion that decreasing the number of cholinergic neurons in this area can cause a disturbance in attention [10].

Potentially, it can be assumed that drugs that demonstrate the inhibition of both AChE and BuChE are preferable. It is difficult to say which substances prove to be more efficient. Certainly, we know that irreversible AChE inhibitors can cause serious toxicity and may even lead to death; hence, only reversible inhibitors are of therapeutic use [3,5].

There are also opinions in publications suggesting that compounds selectively inhibiting BuChE will be more effective than selective AChE inhibitors. These findings are based on the published results of work relating to the activity of huperzine A and the analysis of the effects of inhibitors presently used in medicine [11]. Galanthamine (**1**) and donepezil are reversible inhibitors of both cholinesterases (transient bonding), while rivastigmine is pseudo-irreversible (covalent bonding with the enzyme). Hence, a greater focus on selective inhibitors of BuChE is suggested [12].

The purpose of this review is to provide updated information (from the last 15 years) on cholinesterase inhibitors present in plant materials, discuss their structure–activity correlation and describe methods that can be used for their analysis. We hope that such a comprehensive review will serve as a guide for scientists willing to find potentially novel molecules for neurodegenerative disorders, such as AD. 

## 2. Chemistry–Structure Activity

Cholinesterase inhibitors belong to different groups of compounds. It may be noted here that compounds that have shown activity generally are in similar classes and included in the same group of compounds or even the same type in the group.

Compounds of natural origin showing an inhibitory effect on cholinesterase (ChE) can be considered in terms of the potency of their activity, their selectivity for each cholinesterase or their method of binding to the enzyme (reversible, pseudo-irreversible or irreversible inhibitors) [13].

The ability to inhibit cholinesterases is observed in various groups, including alkaloids, anthranoids, bibenzyls, coumarins, chromones, diarylheptanoids, fatty acids, flavonoids, lignans, phenanthrenes, phenylpropanoids, phthalates, phenolic acids, phlorotannins, polyphenols, polyketides, steroids (sterols), terpenes (diterpenes, triterpenoids, lanostane triterpenes and sesquiterpenes (sesquiterpene lactones)), stilbenoids, triflavanones and xanthonoids.

The majority of hitherto known, applied and potent IChEs (e.g., galanthamine (**1**), rivastigmine and tacrine) are derived from the group of alkaloids. Additionally, flavonoids and coumarins (IBuChE) have become increasingly important as appropriate inhibitors, because they show strong inhibition of ChE and fewer side effects. 

Therefore, the focus is on describing the correlation between the activity and structure of selected groups of compounds for which the results of AChE or BuChE inhibition were the most promising (Table 1) [13]. There are reports that suggest a correlation of the activity of a compound on AChE and BuChE with certain components of its structure.

### 2.1. Alkaloids

These metabolites are characterized by the presence of nitrogen in a negative oxidation state (proton acceptor), in most cases positioned in a heterocycle. This may affect the active site of cholinesterase [13,14].

Because of its use in therapeutics, galanthamine (**1**) may be considered the most important alkaloid inhibiting cholinesterases. It is applied in AD treatment or other neurological disorders. Amaryllidaceae plants are natural sources of galanthamine (**1**). Some species of *Narcissus*, *Leucojum* and *Ungernia* genera are particularly rich in this alkaloid. It can also be obtained synthetically. There were also attempts to obtain it through biosynthesis [15].

Galanthamine (**1**) has a strong inhibitory effect on both AChE and BuChE; however, it is more selective toward AChE. It reveals competitive inhibition; additionally, it has a modulating impact on the nicotinic acetylcholine receptor. Thanks to this effect, it also supports neuromuscular conduction [15,16,17]. There are many publications describing the inhibition of cholinesterase by galanthamine (**1**). Thus, it is often treated as a reference substance (Table 1). On the basis of research on the interaction between galanthamine (**1**) and AChE from *Torpedo californica*, it was found to bind in the active center of the enzyme. The interaction between the double bond present in the galanthamine (**1**) cyclohexene ring and Trp84 enzyme was observed [18].

**Figure 1:**
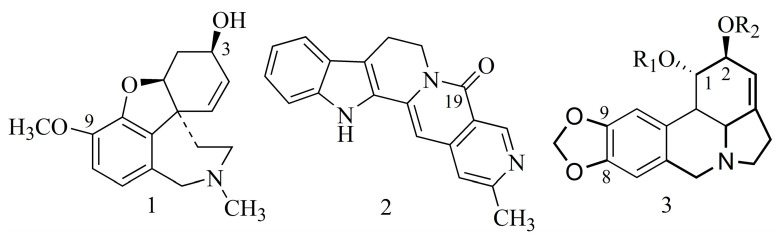
Chemical structures of galanthamine (**1**) and angustidine (**2**) and general structure of lycorine-type alkaloids (**3**).

Monoterpenoid indole alkaloids from *Nauclea officinalis* exhibit inhibitory activity against BuChE. The inhibitory impact of some of them (Table 1, Figure 1) is greater than that of galanthamine (**1**) [19]. Liew et al. (2015) [19], after performing molecular docking, speculate that the high value of cholinesterase inhibition exhibited by angustidine (**2**) is due to the hydrogen bonding (atom C-19 participates in the hydrogen bond) of the inhibitor with amino acids of the enzyme (Ser 198 and His 438) (Figure 1). On the basis of the structure–activity relationship (SAR), McNulty et al. (2010) [18] indicated that the inhibitory effect of lycorine-type alkaloids on AChE is due to an increase in the involvement of the lipophilic substituent in C-1 and C-2 acting as hydroxyl in galanthamine (**1**) (general structure of lycorine-type alkaloids (**3**)) (Figure 1).

**Figure 2:**
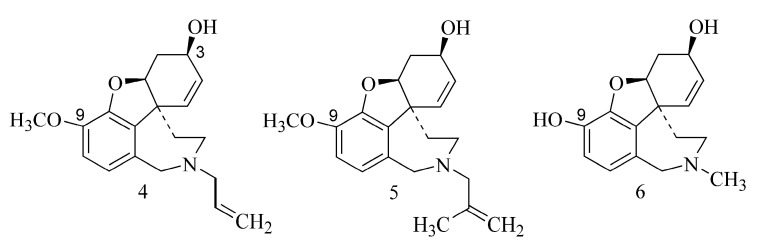
Chemical structures of *N*-allyl-*nor*-galanthamine (**4**), *N*-(14-methylallyl)-*nor*-galanthamine (**5**) and sanguinine (**6**).

According to Berkov et al. (2008), the alkaloids *N*-allyl-*nor*-galanthamine (**4**) and *N*-(14-methylallyl)-*nor*-galanthamine (**5**) isolated from the leaves of *Leucojum aestivum* L. demonstrated more potent inhibition of AChE than galanthamine (**1**) (Table 1). It appears that the inhibitory activity of both compounds is due to the substitution of the *N*-methyl derivative (allyl or 14-methylallyl group). The compounds are characterized by the presence of a methoxyl substituent at C-9, and the nitrogen atom also has a substituent alkyl group (Figure 2), which may indicate its greater lipophilicity compared to galanthamine (**1**) [20]. Among the alkaloids belonging to the Amaryllidaceae family (Table 1), sanguinine (**6**) isolated from *Galanthus woronowii* or *Hieronymiella marginata* [21,22] is the most potent. It is also substituted at the N atom but with a methyl group; however, this is the same moiety as in the case of galantamine. The stronger activity of sanguinine (**6**) compared to galanthamine (**1**), *N*-allyl-*nor*-galanthamine (**4**) and *N*-(14-methylallyl)-*nor*-galanthamine (**5**) may be explained by the presence of a hydroxyl group at the C-9 carbon and is not due to a methoxy group as in their case. The stronger the directing effect of the hydroxyl substituent (compared to the methoxy group), the stronger the activation of the aromatic ring in the electrophilic substitution reaction (Figure 2).

The structures of isoquinoline alkaloids of the protoberberine type (Table 1) are similar to the structure of acetylcholine, containing an anionic site—acetoxy—and simultaneously a cationic site (amine). As in the case of acetylcholine, this structure may enable the bonding of the acetoxy group to the serine hydroxyl group at the site of hydrolysis of the substrate located in the esteratic site of AChE. The cationic site may be an isoquinoline nitrogen atom [1]. Protoberberine-type alkaloids (e.g., berberine (**7**), dihydroberberine (**8**) and coptisine (**9**)) such as Amaryllidaceae alkaloids are characterized by the presence of substituent methoxy and hydroxy groups or methylenedioxy groups, but in different positions (at C-2, C-3 and C-9, C-10), as well as a positively charged nitrogen atom [23].

**Figure 3:**
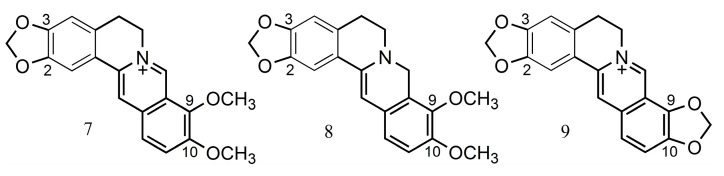
Chemical structures of berberine (**7**), dihydroberberine (**8**) and coptisine (**9**).

As noted by Song et al. (2021), the presence of a conjugated aromatic system in the B ring is responsible for the strong inhibitory activity (e.g., berberine (**7**), coptisine (**9**), epiberberine, jatrorrhizine and palmatine (Table 1)). The hydrogenation of this ring decreases the inhibitory activity of the alkaloid (e.g., dihydroberberine (**8**)), while the cyclization leading to the methylenedioxy group has no impact on this activity (e.g., coptisine (**9**)) [23] (Figure 3).

**Figure 4:**
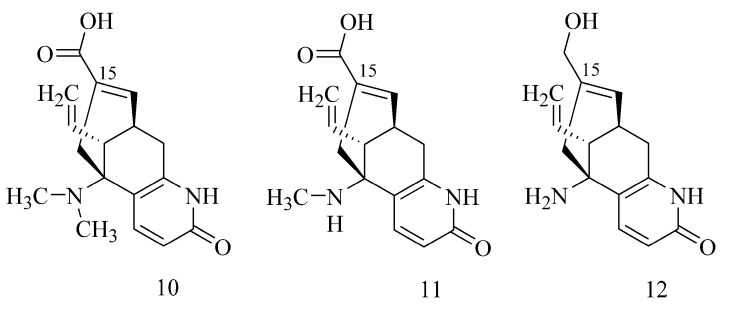
Chemical structures of lycoparin A (**10**), lycoparin B (**11**) and lycoparin C (**12**).

In the case of alkaloids extracted from *Lycopodium casuarinoides* (lycoparins A (**10**), B (**11**) and C (**12**)), the structure is also important in the inhibitory activity. Only lycoparin C (**12**) showed such an ability (Table 1), whereas lycoparins A (**10**) and B (**11**) have poor activity (IC_50_ > 200 µM) as a consequence of the occurrence of carboxylic acid at the C-15 and methyl substituents attached to N (Figure 4) [24].

**Figure 5:**
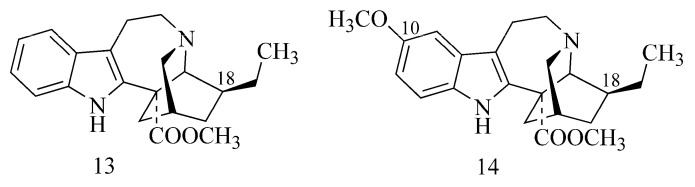
Chemical structures of coronaridine (**13**) and voacangine (**14**).

Strong inhibitory activity against AChE comparable to that of galanthamine (**1**) is demonstrated by indole alkaloids from *Ervatamia hainanensis* (coronaridine (**13**) and voacangine (**14**)). Due to the presence of the substituent voacangine (14), they have markedly increased AChE inhibition. This is because of the attachment of the methoxyl substituent to the phenyl group, while the substitution of 10-hydroxycoronaridine with a hydroxy group on the phenyl decreases the activity (Table 1) (Figure 5) [25].

### 2.2. Coumarins

Coumarins are derivatives of an α-pyrone ring fused with benzene. Hydroxycoumarin (a hydroxyl group), methoxycoumarin (a methoxy group) (substituted at C-7, C-5 or less so at C-6, C-8), furanocoumarin (a furan ring) and piranocoumarin (a pyran ring) have been distinguished. 

Research on the structure and inhibition led to the conclusion that furanocoumarins have more affinity for BuChE than AChE [13,14]. Cholinesterase-inhibiting coumarins are often found in the Apiaceae and Rutaceae families [26].

**Figure 6:**
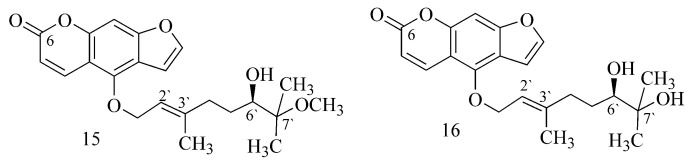
Chemical structures of 6′-hydroxy-7′-methoxybergamottin (**15**) and 6′,7′- dihydroxybergamottin (**16**).

It is noted that the effect of compounds isolated from an extract of *Citrus hystrix* (6′-hydroxy-7′-methoxybergamottin (**15**) and 6′, 7′-dihydroxybergamottin (**16**)) against BuChE depends on the presence of a dioxygenated geranyl chain in their structures (Figure 6) [27].

**Figure 7:**
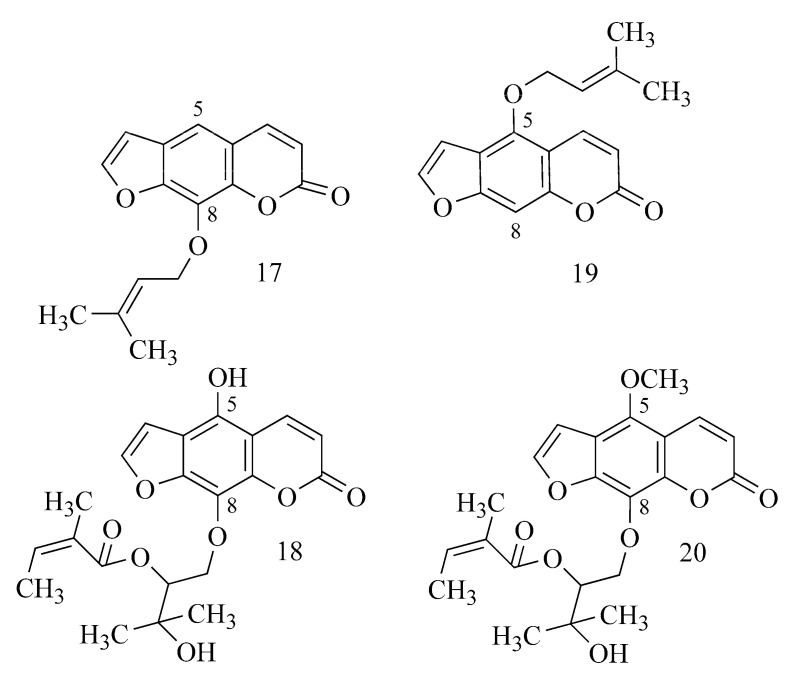
Chemical structures of imperatorin (**17**), heraclenol-2′-O-angelate (**18**), isoimperatorin (**19**) and byakangelicin-2′-O-angelate (**20**).

In a study of the activity of coumarins from *Angelica archangelica* L., the authors assume that BuChE inhibitory activity occurs only in C-8-substituted furanocoumarins (imperatorin (**17**), heraclenol-2′-O-angelate (**18**) (Table 1)). Simple coumarins (osthole and archangelicin), 5-substituted furanocoumarins (isoimperatorin (**19**), phellopterin, bergapten and isopimpinellin) and substituted derivatives at both C-5 and C-8 (byakangelicin-2′-O-angelate (**20**) and byakangelicin-2′-O-isovalerate) do not show this effect (Figure 7) [28].

**Figure 8:**
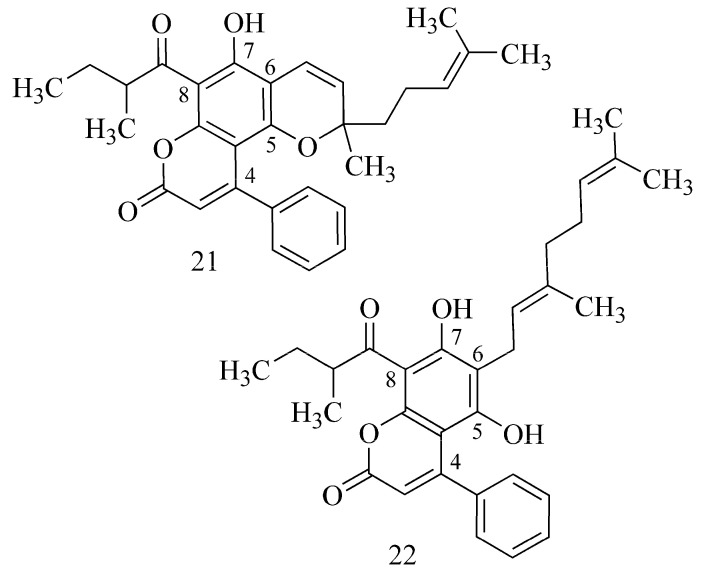
Chemical structures of mesuagenin B (**21**) and 5,7-dihydroxy-8-(3-methylbutanoyl)-6-[(*E*)-3,7-dimethylocta-2,6-dienyl]-4-phenyl-2H-chromen-2-one (**22**). (*Stereochemistry not determined).

Compounds isolated from *Mesua elegans* such as 4-phenylcoumarins [29] show an explicit impact of inhibiting of AChE, because the activity increases for those which contain a dimethylpyran ring at C-5/C-6 and a prenyl substituent in position C-3 (mesuagenin B (**21**)). For 6-geranylated coumarins (5,7-dihydroxy-8-(3-methylbutanoyl)-6-[(*E*)-3,7-dimethylocta-2,6-dienyl]-4-phenyl-2H-chromen-2-one (**22**)), the activity increases in the case of the presence of a 2-methylbutanoyl group, and it is lower for those with a 2-methylpropanoyl or 3-methylbutanoyl group at C-8 (Figure 8) [29].

### 2.3. Diarylheptanoids

Diarylheptanoids are a group of natural compounds with structures based on a 1,7-diphenylheptane skeleton [30].

**Figure 9:**
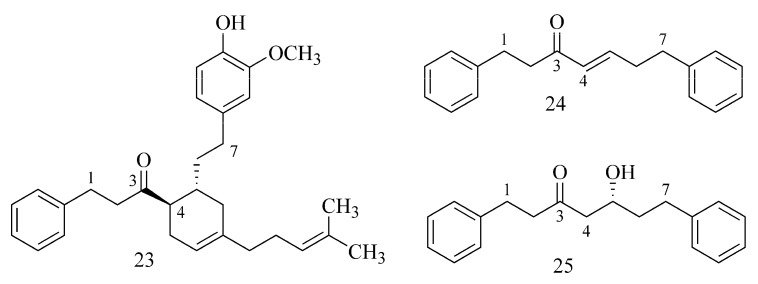
Chemical structures of (−)-alpininoid B (**23**), (4*E*)-1,7-diphenyl 4-hepten-3-one (**24**) and dihydroyashsbushiketol (**25**).

In diarylheptanoids isolated from *Alpinia officinalis* by Lee et al. (2018) [31] (Table 1), it has been observed that the ChE inhibition strength is related to the presence of double bonds in the molecule and is proportional to their number. Thus, (−)-alpininoid B (**23**) exhibits the strongest AChE and BuChE inhibition, whereas (4*E*)-1,7 diphenyl 4-hepten-3-one is weaker (**24**), and dihydroyashsbushiketol is the weakest (**25**), where additional bonds are absent (Figure 9) [31].

### 2.4. Flavonoids

**Figure 10:**
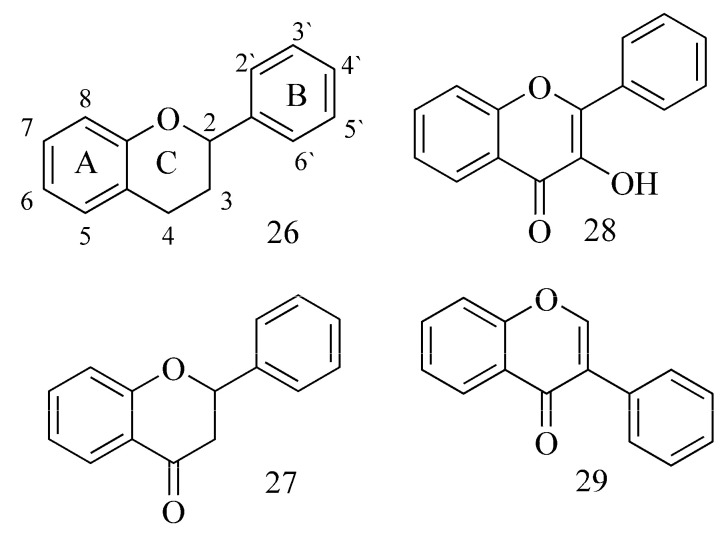
General chemical structures of flavonoid (**26**), flavanone (**27**), flavonol (**28**) and isoflavone (**29**) rings.

Flavonoids are highly active inhibitors with low toxicity [29]. The flavonoid group consists of flavanones (**27**), flavonols (**28**), dihydroflavonols, flavones, isoflavones (**29**), chalcones, dihydrochalcones and aurones (Figure 10) [14].

The bond-line formula of flavonoids is made of two aromatic rings linked to diphenylpropane in a C6-C3-C6 system. Most of them have an additional gamma-pyrone system (rings C) divided into types due to the different positions of the B ring, the oxidation number of the C ring and the presence of additional functional groups [13,14,32].

Xie et al. (2014) [32] studied the link between the binding affinities of flavonoids with AChE using a typical measurement—the fluorescence quenching method reported by Ryu et al. (2012) [33]. They checked 20 flavonoids (i.e., baicalin, genistein, chrysin, apigenin, formononetin, 7,8-dihydroflavone, puerarin, luteolin, rutin (**36**), fisetin, naringenin, daidzein, daidzin, myricetin, myricetrin, quercetin, quercetrin, kaempferol (**35**), kaempferide and baicalein). According to this research, it can be inferred that inhibitory flavonoids form a complex with AChE. The presence of a hydroxyl group, especially in the A ring of the flavonoid, as well as the double bond between C-2 and C-3, increases the affinity of the enzyme (hydrogen bonds) and also increases the AChE inhibitory properties of flavonoids. Glycosylation, on the other hand, decreases the activity and affinity of flavonoids toward the enzyme in a manner that depends on the form of the attached sugar moiety (1–5-fold). The presence of a methoxy group affects the activity of a flavonoid differently depending on its type, and no correlation was observed here [32].

**Figure 11:**
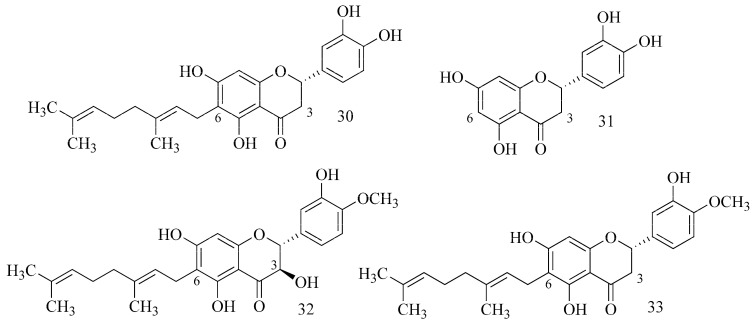
Chemical structures of diplacone (**30**), eriodictyol (**31**)**,** 4′-O-methyldiplacol (**32**) and 4′-O-methyldiplacone (**33**).

Analyzing the impact of the structure of flavonoids from *Paulownia tomentosa* fruits indicated that geranylated flavonoids at C-6 (e.g., diplacone (**30**)) (Table 1) are pivotal against hAChE and BuChE. The lack of this moiety causes a clear decrease in inhibition (eriodictyol (**31**) (IC_50_ = 1663 µM)). It has also been proved that dihydroflavonols (4′-O-methyldiplacol (**32**)) show stronger inhibition compared to flavones (4′-O-methyldiplacone (**33**)) (Figure 11) [34].

**Figure 12:**
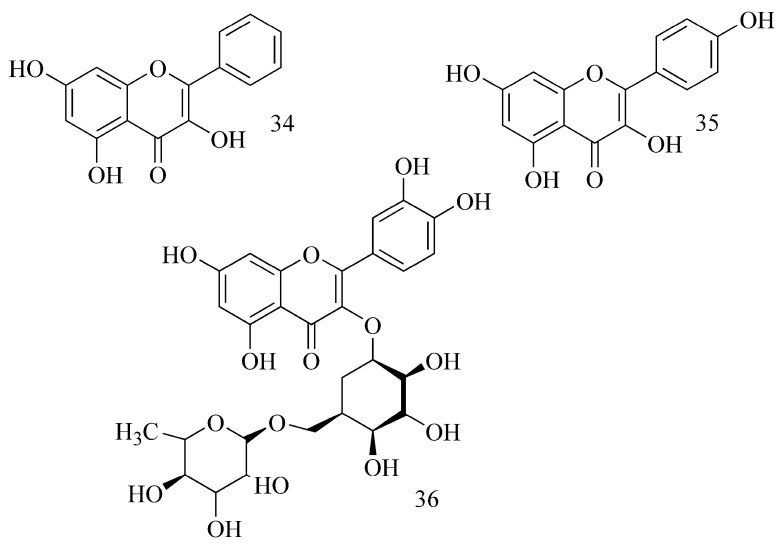
Chemical structures of galangin (**34**), kaempferol (**35**) and rutin (**36**).

Selected flavonoids have been studied (docking study) (galangin (**34**), kaempferol (**35**), quercetin, myricetin, fisetin, apigenin, luteolin and rutin (**36**)) [35]. The inhibitory potency of flavonoids toward BuChE depends on the presence and the location of OH groups in the structure. A sugar moiety causing steric hindrance reduces these properties. Galangin (**34**) showed the strongest activity, kaempferol (**35**) was proved to be weaker, and rutin (**36**) was the weakest (Figure 12).

### 2.5. Phenanthrenes

Phenanthrenes are a group of natural compounds with a structure based on the phenanthrene skeleton, occurring in the form of monomeric, dimeric or trimeric derivatives [36].

**Figure 13:**
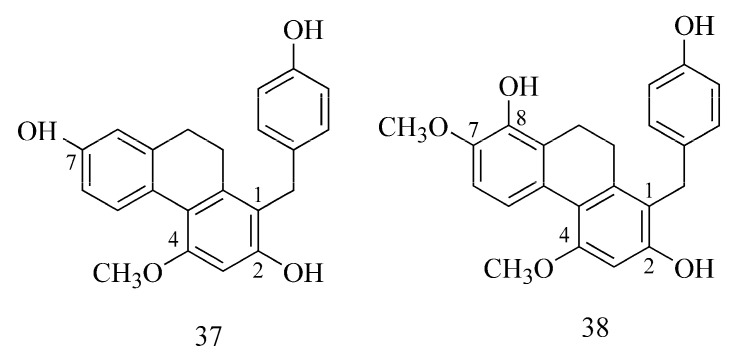
Chemical structures of 1-[(4-hydroxyphenyl)methyl]-4-methoxy-2,7-phenanthrenediol (**37**) and 1-(4-hydroxybenzyl)-4, 7-dimethoxyphenanthrene-2,8-diol (**38**).

Phenanthrenes from *Bletilla striata* showed potent and selective inhibitory activity against BuChE [37]. A publication by Liu et al. (2022) described that the presence of substituents at C-2 and C-7 is responsible for the stronger BuChE inhibition of phenanthrenes from *Bletilla striata*. The activity is more potent when the phenanthrene is substituted with a hydroxy group (e.g., 1-[(4-hydroxyphenyl)methyl]-4-methoxy-2,7-phenanthrenediol (**37**)), while substitution with a methoxy group reduces this effect (e.g., 1-(4-hydroxybenzyl)-4, 7-dimethoxyphenanthrene-2,8-diol (**38**)). Substituents at C-8 (hydroxy group) and also at C-1 (4-hydroxybenzyl) improve the affinity to the enzyme (Figure 13) [37].

### 2.6. Terpenes

These are compounds aggregated from properly bound isoprene subunits. We can distinguish monoterpenes, sesquiterpenes, diterpenes and triterpenes [14].

**Figure 14:**
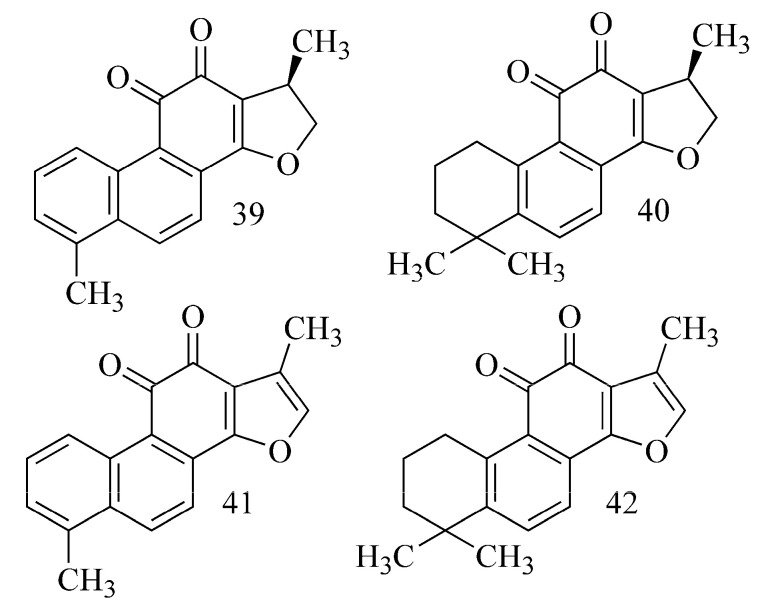
Chemical structures of dihydrotanshinone I (**39**), cryptotanshinone (**40**), tanshinone I (**41**) and tanshinone IIA (**42**).

By testing acetone extracts of the roots of *Salvia miltiorhiza* Bunge, strong inhibitory activity against AChE for the diterpenes dihydrotanshinone I (**39**) (IC_50_ = 1 μM) and cryptotanshinone (**40**) (IC_50_ = 7 μM) and weak activity for tanshinone I (**41**) (IC_50_ > 50 μM) and tanshionone IIA (**42**) (IC_50_ > 140 μM) [38] (Table 1) were found by Ren et al. (2004). The authors suppose that the activity is probably a result of the existence of a dihydrofuran ring instead of a furan ring present in the compounds indicating weak inhibitory activity. Additionally, compounds containing an aromatic ring in their structures showed much higher activity than those that have a cyclohexane ring at this site [38]. However, the study by Zhou et al. (2011) showed quite different results [39]. Inhibitory activity was not observed in tanshinone IIA (**42**) or cryptotanshinone (**40**), but tanshinone I (**41**) and dihydrotanshinone I (**39**) showed strong activity. Both of these compounds are similar in terms of o-aromatic rings; they only differ in the presence or lack of a double bond in the furan ring. The authors suggest that for the inhibitory effect on AChE, the structure of the aromatic ring may be more important than the furan ring as was thought before (the presence or lack of a double bond) (Figure 14) [39].

### 2.7. Xanthonoids

Xanthonoids and xanthones are subgroups of polyphenols with structures based on the tricyclic skeleton dibenzo-γ-pirone [40].

In the study by Urbain et al. (2004), xanthones isolated from *Gentiana campestris* exhibited inhibitory activity against AChE [41]. Bellidifolin (**43**) had the best result. It achieved a minimum inhibitory quantity on TLC identical to that of galanthamine (**1**) (0.03 nM), while weaker results were those of bellidin (**44**) (0.15 nM) and its bellidifolin glycosides: 8-O-β-glucopyranoside (*nor*-swertianolin) and 8-O-β-glucopyranoside (swertianolin) were even weaker (0.18 and 1.2 nM, respectively) [41]. The weaker inhibition of the enzyme by glycosides can probably be explained by steric hindrance and diverted hydrophobicity. On the other hand, xanthones containing an additional methoxyl group in the C-3 position showed stronger activity [41].

**Figure 15:**
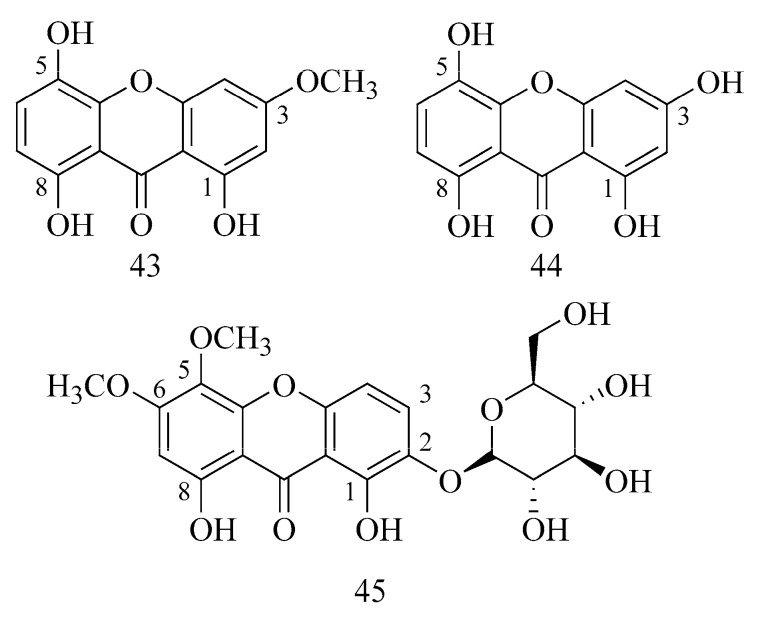
Chemical structures of bellidifolin (**43**), bellidin (**44**) and triptexanthoside C (**45**).

In a more recent study by Urbain et al. (2008), the activity of xanthones of *Gentianella amarella* ssp. *acuta* was examined [42]. They exhibited weaker activity (also including bellidin (**44**) and bellidifolin (**43**)), and only triptexanthoside C (**45**) reached significant results for activity against AChE (Table 1) [42]. This compound also has a methoxyl group in its structure, which may influence the higher result of cholinesterase inhibition (Figure 15).

In summary, the potential activity of an acetylcholinesterase inhibitor is influenced by the presence of hydroxyl and methoxyl groups in the molecule and also by the presence of the cationic part of the structure of the compound (e.g., nitrogen in the heterocyclic system). The substrate-like structure of the inhibitor (or acetylcholine) indicates the competitive inhibition of the enzyme, and it is most beneficial in pharmacology. Large molecules, e.g., glycosidic forms of the tested compounds, were characterized by weaker AChE inhibitory activity due to their steric hindrance in the enzyme. The occurrence, different number and localization of double bonds, preferably in conjugated systems (diarylheptanoids and Amaryllidaceae alkaloids), are of utmost importance. With the increase in the number of conjugated double-bond systems, as well as the presence of substituents that polarize the aromatic system, the energy of the cation–π interaction increases, and thus, the binding energy of the inhibitor with the protein residue of the enzyme increases [43]. The presence of these substituents in the compounds was also significant in the inhibition against AChE. This may be related to the ability of BuChE to hydrolyze both butyrylcholine and acetylcholine [1,44]. The structure of the BuChE enzyme molecule enables the catalysis of large acyl groups, which the AChE molecule is not capable of. Hence, in the presented data (Table 1), there are many inhibitors that are inactive against AChE while demonstrating moderate or strong activity toward BuChE [1]. This may be due to the steric hindrance of the AChE enzyme due to the large branched structures of such compounds, as is demonstrated by the weaker activity of glycosides in relation to their aglycones (xanthonoids from *Gentiana campestris*) (Table 1).

The review topic of natural cholinesterase inhibitors has been discussed in other publications, including [45,46,47]. Most of them are based on the description of results obtained for plant fractions and extracts or, in addition, for compounds isolated from them [45,46]. This article focuses on the comparison of particular isolated natural compounds’ activities, considering both plant and animal origins (e.g., alkaloids from scorpions or sponges). Some of the previous reviews did not include this information [45,46]. The current review includes 20 groups (24 subgroups) of compounds; a total of 357 results for cholinesterase inhibition by natural compounds are listed, arranged alphabetically by compound group, species name and compound name. A total of 84 species or their varieties belonging to 44 families were examined. The current review shows, in tabular form, the results of the inhibition of both AChE and BuChE enzymes. The present summary is also characterized by the fact that the type of enzyme and the method used in the study are presented. This review shows that differences are significant and have an impact on the results of enzyme inhibition by the tested compounds. This paper focuses on the review of the results of studies on natural cholinesterase inhibitors tested using in vitro methods. The presented overview is also characterized by the description and consideration of the type of method used for the determination of cholinesterase inhibition, which has not been undertaken in other recent reviews, or they were limited to the modifications of colorimetric Ellman’s method [46].

The data, mainly from the selected latest publications issued from 2008 to 2022, on cholinesterase inhibitors of natural origin are ordered in the table below (Table 1). The following sources were used to prepare the review article database: Chemical Abstract (SciFinder), Reaxys and Science Direct (partially by authorized access), as well as sources directly obtained from the authors (ResearchGate GmbH)).

**Table 1:** Inhibitors’ classification in terms of their affiliation with a group of compounds, their effects on AChE and BuChE, their activity, their origins and the methods of their analysis.

Inhibitors	Source	Activity				Method	Ref.
		Value of Inhibition against AChE	Reference Standard for AChE	Value of Inhibition against BuChE	Reference Standard for BuChE		
**ALKALOIDS**							
Lindoldhamine isomer	*Abuta panurensis* Eichler Menispermaceae (branches)	39.38 ± 0.08 µM ^a,j^	NEO 3.72 ± 0.03 µM ^a,j^	nd	nd	MCE	[48,49,50]
5-*N*-Methylmaytenine	*Abuta panurensis* Eichler Menispermaceae (branches)	19.55 ± 0.09 µM ^a,j^	NEO 3.72 ± 0.03 µM ^a,j^	nd	nd	MCE	
*N*-trans-Feruloyltyramine	*Abuta panurensis* Eichler Menispermaceae (branches)	na	NEO 3.72 ± 0.03 µM ^a,j^	nd	nd	MCE	
Palmatine	*Abuta panurensis* Eichler Menispermaceae (branches)	35.25 ± 0.04 µM ^a,j^	NEO 3.72 ± 0.03 µM ^a,j^	nd	nd	MCE	
Stepharine	*Abuta panurensis* Eichler Menispermaceae (branches)	61.24 ± 0.03 µM ^a,j^	NEO 3.72 ± 0.03 µM ^a,j^	nd	nd	MCE	
Aconorine	*Aconitum laeve* Ranunculaceae (tubers)	2.51 ± 0.037 µM ^a,e^	GAL 3.26 ± 0.021 µM ^a,e^	8.72 ± 0.023 µM ^a,m^	GAL 10.13 ± 0.05 µM ^a,m^	MCE	[51,52]
Hohenackerine	*Aconitum laeve* Ranunculaceae (tubers)	4.53 ± 0.062 µM ^a,e^	GAL 3.26 ± 0.021 µM ^a,e^	9.94 ± 0.073 µM ^a,m^	GAL 10.13 ± 0.05 µM ^a,m^	MCE	
Lappaconotine	*Aconitum laeve* Ranunculaceae (tubers)	6.13 ± 0.019 µM ^a,e^	GAL 3.26 ± 0.021 µM ^a,e^	11.24 ± 0.12 µM ^a,m^	GAL 10.13 ± 0.05 µM ^a,m^	MCE	
Swatinine-C	*Aconitum laeve* Ranunculaceae (tubers)	3.7 ± 0.085 µM ^a,e^	GAL 3.26 ± 0.021 µM ^a,e^	12.23 ± 0.014 µM ^a,m^	GAL 10.13 ± 0.05 µM ^a,m^	MCE	
4-Methoxy-1-methyl-2-quinolone	*Atractylis cancellata* L. Asteraceae (whole plant)	>50 µg mL^−1 a,k^	GAL 6.27 ± 1.15 µg mL^−1 a,k^	37.49 ± 1.61 µg mL^−1 a,n^	GAL 34.75 ± 1.99 µg mL^−1 a,n^	MCE	[53]
Pyrroloquinolone A	*Atractylis cancellata* L. Asteraceae (whole plant)	18.48 ± 0.33 µg mL^−1 a,k^	GAL 6.27 ± 1.15 µg mL^−1 a,k^	9.66 ± 0.16 µg mL^−1 a,n^	GAL 34.75 ± 1.99 µg mL^−1 a,n^	MCE	
Buthutin A	*Buthus martensii* Karsch Buthidae (whole body of scorpion)	7.83 *±* 0.06 µM ^a,e^	GAL 1.17 ± 0.01 µM ^a,e^ DON 0.049 ± 0.004 µM ^a,e^	47.44 *±* 0.95 µM ^a,m^	GAL 18.78 ± 1.81 µM ^a,m^ DON 5.536± 0.018 µM ^a,m^	MCE	[48,54,55]
Buthutin B	*Buthus martensii* Karsch Buthidae (whole body of scorpion)	61.45 ± 2.34 µM ^a,e^	GAL 1.17 ± 0.01 µM ^a,e^ DON 0.049 ± 0.004 µM ^a,e^	122.64 *±* 5.21 µM ^a,m^	GAL 18.78 ± 1.81 µM ^a,m^ DON 5.536± 0.018 µM ^a,i^	MCE	
Trigonelline	*Buthus martensii* Karsch Buthidae (whole body of scorpion)	97.30 ± 4.18 µM ^a,e^	GAL 1.17 ± 0.01 µM ^a,e^ DON 0.049 ± 0.004 µM ^a,e^	441.87 ± 7.99 µM ^a,m^	GAL 18.78 ± 1.81 µM ^a,m^ DON 5.536± 0.018 µM ^a,m^	MCE	
17-*oxo*-3-Benzoylbuxadine	*Buxus hyrcana* Pojark. Buxaceae (leaves)	17.6 ± 0.5 µM ^a,k^	GAL 0.53 ± 0.5 µM ^a,k^ HUP 1.7 ± 0.3 µM ^a,k^	186.8 ± 1.0 µM ^a,n^	GAL 8.7 ± 1.0 µM ^a,n^ HUP >1000 ± 3.0 µM ^a,n^	MCE	[48,56,57,58]
31-Demethylcyclobuxoviridine	*Buxus hyrcana* Pojark. Buxaceae (leaves)	298.3 ± 1.0 µM ^a,k^	GAL 0.53 ± 0.5 µM ^a,k^ HUP 1.7 ± 0.3 µM ^a,k^	15.4 ± 0.5 µM ^a,n^	GAL 8.7 ± 1.0 µM ^a,n^ HUP >1000 ± 3.0 µM ^a,n^	MCE	
31-Hydroxybuxamine B	*Buxus hyrcana* Pojark. Buxaceae (leaves)	61.3 ± 2.0 µM ^a,k^	GAL 0.53 ± 0.5 µM ^a,k^ HUP 1.7 ± 0.3 µM ^a,k^	112.1 ± 3.0 µM ^a,n^	GAL 8.7 ± 1.0 µM ^a,n^ HUP >1000 ± 3.0 µM ^a,n^	MCE	
Buxamine A	*Buxus hyrcana* Pojark. Buxaceae (leaves)	81.4 ± 2.4 µM ^a,k^	GAL 0.53 ± 0.5 µM ^a,k^ HUP 1.7 ± 0.3 µM ^a,k^	100.2 ± 1.4 µM ^a,n^	GAL 8.7 ± 1.0 µM ^a,n^ HUP >1000 ± 3.0 µM ^a,n^	MCE	
Buxamine B	*Buxus hyrcana* Pojark. Buxaceae (leaves)	79.6 ± 3.0 µM ^a,k^	GAL 0.53 ± 0.5 µM ^a,k^ HUP 1.7 ± 0.3 µM ^a,k^	100.5 ± 2.5 µM ^a,k^	GAL 8.7 ± 1.0 µM ^a,n^ HUP >1000 ± 3.0 µM ^a,n^	MCE	
Buxhyrcamine	*Buxus hyrcana* Pojark. Buxaceae (leaves)	18.2 ± 0.3 µM ^a,k^	GAL 0.53 ± 0.5 µM ^a,k^ HUP 1.7 ± 0.3 µM ^a,k^	209.0 ± 1.0 µM ^a,n^	GAL 8.7 ± 1.0 µM ^a,n^ HUP >1000 ± 3.0 µM ^a,n^	MCE	
Buxmicrophylline F	*Buxus hyrcana* Pojark. Buxaceae (leaves)	22.4 ± 0.7 µM ^a,k^	GAL 0.53 ± 0.5 µM ^a,k^ HUP 1.7 ± 0.3 µM ^a,k^	154.2 ± 1.0 µM ^a,n^	GAL 8.7 ± 1.0 µM ^a,n^ HUP >1000 ± 3.0 µM ^a,n^	MCE	
Buxrugulosamine	*Buxus hyrcana* Pojark. Buxaceae (leaves)	24.8 ± 1.0 µM ^a,k^	GAL 0.53 ± 0.5 µM ^a,k^ HUP 1.7 ± 0.3 µM ^a,k^	160.2 ± 4.0 µM ^a,n^	GAL 8.7 ± 1.0 µM ^a,n^ HUP >1000 ± 3.0 µM ^a,n^	MCE	
Cyclobuxophylline O	*Buxus hyrcana* Pojark. Buxaceae (leaves)	35.4 ± 1.0 µM ^a,k^	GAL 0.53 ± 0.5 µM ^a,k^ HUP 1.7 ± 0.3 µM ^a,k^	45.0 ± 2.0 µM ^a,n^	GAL 8.7 ± 1.0 µM ^a,n^ HUP >1000 ± 3.0 µM ^a,n^	MCE	
Cyclobuxoviridine	*Buxus hyrcana* Pojark. Buxaceae (leaves)	179.7 ± 0.4 µM ^a,k^	GAL 0.53 ± 0.5 µM ^a,k^ HUP 1.7 ± 0.3 µM ^a,k^	304.5 ± 1.0 µM ^a,n^	GAL 8.7 ± 1.0 µM ^a,n^ HUP >1000 ± 3.0 µM ^a,n^	MCE	
*E*-Buxenone	*Buxus hyrcana* Pojark. Buxaceae (leaves)	71.0 ± 2.5 µM ^a,k^	GAL 0.53 ± 0.5 µM ^a,k^ HUP 1.7 ± 0.3 µM ^a,k^	200.7 ± 2.6 µM ^a,n^	GAL 8.7 ± 1.0 µM ^a,n^ HUP >1000 ± 3.0 µM ^a,n^	MCE	
Homomoenjodarmine	*Buxus hyrcana* Pojark. Buxaceae (leaves)	19.5 ± 1.0 µM ^a,k^	GAL 0.53 ± 0.5 µM ^a,k^ HUP 1.7 ± 0.3 µM ^a,k^	52.2 ± 3.0 µM ^a,n^	GAL 8.7 ± 1.0 µM ^a,n^ HUP >1000 ± 3.0 µM ^a,n^	MCE	
Moenjodaramine	*Buxus hyrcana* Pojark. Buxaceae (leaves)	25.0 ± 2.9 µM ^a,k^	GAL 0.53 ± 0.5 µM ^a,k^ HUP 1.7 ± 0.3 µM ^a,k^	102.4 ± 2.0 µM ^a,n^	GAL 8.7 ± 1.0 µM ^a,n^ HUP >1000 ± 3.0 µM ^a,n^	MCE	
*N*_b_-Dimethylcyclobuxoviricine	*Buxus hyrcana* Pojark. Buxaceae (leaves)	45.5 ± 0.6 µM ^a,k^	GAL 0.53 ± 0.5 µM ^a,k^ HUP 1.7 ± 0.3 µM ^a,k^	133.8 ± 3.4 µM ^a,n^	GAL 8.7 ± 1.0 µM ^a,n^ HUP >1000 ± 3.0 µM ^a,n^	MCE	
*N*_20_-Formylbuxaminol E	*Buxus hyrcana* Pojark. Buxaceae (leaves)	25.5 ± 0.8 µM ^a,k^	GAL 0.53 ± 0.5 µM ^a,k^ HUP 1.7 ± 0.3 µM ^a,k^	120.9 ± 2.0 µM ^a,n^	GAL 8.7 ± 1.0 µM ^a,n^ HUP >1000 ± 3.0 µM ^a,n^	MCE	
Spirofornabuxine	*Buxus hyrcana* Pojark. Buxaceae (leaves)	6.3 ± 0.6 µM ^a,k^	GAL 0.53 ± 0.5 µM ^a,k^ HUP 1.7 ± 0.3 µM ^a,k^	125.2 ± 1.0 µM ^a,n^	GAL 8.7 ± 1.0 µM ^a,n^ HUP >1000 ± 3.0 µM ^a,n^	MCE	
Papillozine C	*Buxus hyrcana* Pojark. Buxaceae (leaves)	47.8 ± 1.4 µM ^a,k^	GAL 0.53 ± 0.5 µM ^a,k^ HUP 1.7 ± 0.3 µM ^a,k^	35.2 ± 2.0 µM ^a,n^	GAL 8.7 ± 1.0 µM ^a,n^ HUP >1000 ± 3.0 µM µM ^a,n^	MCE	
*Z*-Buxenone	*Buxus hyrcana* Pojark. Buxaceae (leaves)	87.4 ± 1.7 µM ^a,k^	GAL 0.53 ± 0.5 µM ^a,k^ HUP 1.7 ± 0.3 µM ^a,k^	155.8 ± 3.8 µM ^a,n^	GAL 8.7 ± 1.0 µM µM ^a,n^ HUP >1000 ± 3.0 µM µM ^a,n^	MCE	
Dihydroberberine	*Coptis chinensis* Ranunculaceae (rhizomes)	1.18 ± 0.03 µM ^a,e^	BER 1.01 ± 0.01 µM ^a,e^ TAC 0.22 ± 0.004 µM ^a,e^	38.82 ± 0.52 µM ^a,m^	TAC 0.014 ± 0.0043 µM ^a,m^	MCE	[48,59,60]
10-Hydroxy-infractopicrin	*Cortinarius infractus* Berk Cortinariaceae (toadstool)	12.7 ± 0.16 µM ^a,d^	GAL 8.70 ± 0.05 µM ^a,d^ PHY 2.58 ± 0.03 µM ^a,d^	nd < 100 µM ^a,m^	GAL 24.4 ± 2.84 µM ^a,m^ PHY 1.34 ± 0.279 µM ^a,m^	MCE	[16,48,61]
Infractopicrin	*Cortinarius infractus* Berk Cortinariaceae (toadstool)	9.72 ± 0.19 µM ^a,d^	GAL 8.70 ± 0.05 µM ^a,d^ PHY 2.58 ± 0.03 µM ^a,d^	nd < 100 µM ^a,m^	GAL 24.4 ± 2.84 µM ^a,m^ PHY 1.34 ± 0.279 µM ^a,m^	MCE	
(+)-Adlumine	*Corydalis mucronifera* Maxim. Papaveraceae (whole plants)	>100 µM ^a,e^	GAL 1.34 ± 0.11 µM ^a,e^	>100 µM ^a,m^	GAL 6.81 ± 0.60 µM ^a,m^	MCE	[16,48,62,63]
Bicucullinine	*Corydalis mucronifera* Maxim. Papaveraceae (whole plants)	85.89 ± 0.92 µM ^a,e^	GAL 1.34 ± 0.11 µM ^a,e^	59.75 ± 2.40 µM ^a,m^	GAL 6.81 ± 0.60 µM ^a,m^	MCE	
(−)-Corydalisol	*Corydalis mucronifera* Maxim. Papaveraceae (whole plants)	51.12 ± 0.27 µM ^a,e^	GAL 1.34 ± 0.11 µM ^a,e^	>100 µM ^a,m^	GAL 6.81 ± 0.60 µM ^a,m^	MCE	
Demethylcorydalmine	*Corydalis mucronifera* Maxim. Papaveraceae (whole plants)	71.43 ± 0.55 µM ^a,e^	GAL 1.34 ± 0.11 µM ^a,e^	>100 µM ^a,m^	GAL 6.81 ± 0.60 µM ^a,m^	MCE	
6,7-Dimethoxy-2-methyl-1,2,3,4-tetrahydroisoquinoline	*Corydalis mucronifera* Maxim. Papaveraceae (whole plants)	45.70 ± 0.42 µM ^a,e^	GAL 1.34 ± 0.11 µM ^a,e^	>100 µM ^a,m^	GAL 6.81 ± 0.60 µM ^a,m^	MCE	
1-(1,3-Dioxolo [4,5-*g*]isoquinolin-5-yl)-ethanone	*Corydalis mucronifera* Maxim. Papaveraceae (whole plants)	>100 µM ^a,e^	GAL 1.34 ± 0.11 µM ^a,e^	>100 µM ^a,m^	GAL 6.81 ± 0.60 µM ^a,m^	MCE	
*epi*-Coryximine	*Corydalis mucronifera* Maxim. Papaveraceae (whole plants)	92.00 ± 0.19 µM ^a,e^	GAL 1.34 ± 0.11 µM ^a,e^	>100 µM ^a,m^	GAL 6.81 ± 0.60 µM ^a,m^	MCE	
Hendersine B	*Corydalis mucronifera* Maxim. Papaveraceae (whole plants)	14.22 ± 0.34 µM ^a,e^	GAL 1.34 ± 0.11 µM ^a,e^	>100 µM ^a,m^	GAL 6.81 ± 0.60 µM ^a,m^	MCE	
Hydrohydrastinine	*Corydalis mucronifera* Maxim. Papaveraceae (whole plants)	9.13 ± 0.15 µM ^a,e^	GAL 1.34 ± 0.11 µM ^a,e^	>100 µM ^a,m^	GAL 6.81 ± 0.60 µM ^a,m^	MCE	
9-Methyldecumbenine C	*Corydalis mucronifera* Maxim. Papaveraceae (whole plants)	>100 µM ^a,e^	GAL 1.34 ± 0.11 µM ^a,e^	>100 µM ^a,m^	GAL 6.81 ± 0.60 µM ^a,m^	MCE	
Mucroniferanines H	*Corydalis mucronifera* Maxim. Papaveraceae (whole plants)	2.31 ± 0.20 µM ^a,e^	GAL 1.34 ± 0.11 µM ^a,e^	36.71 ± 1.12 µM ^a,m^	GAL 6.81 ± 0.60 µM ^a,m^	MCE	
Mucroniferanines K	*Corydalis mucronifera* Maxim. Papaveraceae (whole plants)	>100 µM ^a,e^	GAL 1.34 ± 0.11 µM ^a,e^	>100 µM ^a,m^	GAL 6.81 ± 0.60 µM ^a,m^	MCE	
Mucroniferanines L	*Corydalis mucronifera* Maxim. Papaveraceae (whole plants)	>100 µM ^a,e^	GAL 1.34 ± 0.11 µM ^a,e^	>100 µM ^a,m^	GAL 6.81 ± 0.60 µM ^a,m^	MCE	
Mucroniferanines M	*Corydalis mucronifera* Maxim. Papaveraceae (whole plants)	>100 µM ^a,e^	GAL 1.34 ± 0.11 µM ^a,e^	>100 µM ^a,m^	GAL 6.81 ± 0.60 µM ^a,m^	MCE	
(+)-Ochotensine	*Corydalis mucronifera* Maxim. Papaveraceae (whole plants)	>100 µM ^a,e^	GAL 1.34 ± 0.11 µM ^a,e^	>100 µM ^a,m^	GAL 6.81 ± 0.60 µM ^a,m^	MCE	
(−)-Ochrobirine	*Corydalis mucronifera* Maxim. Papaveraceae (whole plants)	>100 µM ^a,e^	GAL 1.34 ± 0.11 µM ^a,e^	>100 µM ^a,m^	GAL 6.81 ± 0.60 µM ^a,m^	MCE	
Orientaline	*Corydalis mucronifera* Maxim. Papaveraceae (whole plants)	83.96 ± 1.06 µM ^a,e^	GAL 1.34 ± 0.11 µM ^a,e^	>100 µM ^a,m^	GAL 6.81 ± 0.60 µM ^a,m^	MCE	
1*R*,9*S*,7′*S*-Methylegenine	*Corydalis mucronifera* Maxim. Papaveraceae (whole plants)	>100 µM ^a,e^	GAL 1.34 ± 0.11 µM ^a,e^	>100 µM ^a,m^	GAL 6.81 ± 0.60 µM ^a,m^	MCE	
5,6,7,8-Tetrahydro-1,3-dioxolo [4,5-*g*]isoquinoline	*Corydalis mucronifera* Maxim. Papaveraceae (whole plants)	>100 µM ^a,e^	GAL 1.34 ± 0.11 µM ^a,e^	>100 µM ^a,m^	GAL 6.81 ± 0.60 µM ^a,m^	MCE	
Pseudocoptisine	*Corydalis turtschaninovii* Besser forma *yanhusuo* Papaveraceae (tuber)	12.8 µM ^a,i^	TAC 0,175 µM ^a,i^	nd	nd	MCE	[64]
(−)-Desmethyl*seco*antofine	*Cryptocarya densiflora* BI. Lauraceae (leaves)	201.52 µM ^a,e^	PHY 0.16 µM ^a,e^	166.69 µM ^a,m^	PHY 0.58 µM ^a,m^	MCE	[48,65,66]
(+)-Laurotetanine	*Cryptocarya densiflora* BI. Lauraceae (leaves)	100 µg mL^−1^—17.51 ± 0.68% ^b,e^	nd	100 µg mL^−1^—22.58 ± 0.47 µM ^a,m^	PHY 0.58 µM ^a,m^	MCE	
(+)-*nor*-Nantenine	*Cryptocarya densiflora* BI. Lauraceae (leaves)	205.55 µM ^a,e^	PHY 0.16 µM ^a,e^	94.45 µM ^a,m^	PHY 0.58 µM ^a,m^	MCE	
(+)-Oridine	*Cryptocarya densiflora* BI. Lauraceae (leaves)	100 µg mL^−1^—27.89 ± 0.64% ^b,e^	nd	288.34 µM ^a,m^	PHY 0.58 µM ^a,m^	MCE	
2-Methoxyatherosperminine	*Cryptocarya griffithiana* Wight. Lauraceae (bark)	100 µg mL^−1^—31.58 ± 2.87% ^b,e^	nd	3.95 µM ^a,m^	PHY 0.58 µM ^a,m^	MCE	
(+)-Reticuline	*Cryptocarya griffithiana* Wight. Lauraceae (bark)	301.01 µM ^a,e^	PHY 0.16 µM ^a,e^	65.04 µM ^a,m^	PHY 0.58 µM ^a,m^	MCE	
Atherosperminine	*Cryptocarya infectoria* Miq. Lauraceae (bark)	100 µg mL^−1^—2.06 ± 1.29% ^b,e^	nd	19.34 µM ^a,m^	PHY 0.58 µM ^a,m^	MCE	
(+)-*N*-Methylisococlaurine	*Cryptocarya infectoria* Miq. Lauraceae (bark)	100 µg mL^−1^—14.93 ± 0.53% ^b,e^	nd	100 µg mL^−1^—37.33 ± 1.56 ^a,m^	PHY 0.58 µM ^a,m^	MCE	
(+)-*N*-Methyllaurotetanine	*Cryptocarya infectoria* Miq. Lauraceae (bark)	100 µg mL^−1^—38.79 ± 2.6% ^b,e^	nd	218.81 µM ^a,m^	PHY 0.58 µM ^a,m^	MCE	
Chitralinine A	*Delphinium chitralense* H. Riedl in Kew Bull. Ranunculaceae (aerial parts)	13.86 ± 0.35 µM ^a,e^	GAL 10.12 ± 0.06 µM ^a,e^ ALA 8.23 ± 0.01 µM ^a,e^	28.17 ± 0.92 µM ^a,m^	GAL 20.62 ± 0.08 µM ^a,m^ ALA 18 ± 0.06 µM ^a,m^	MCE	[48,67]
Chitralinine B	*Delphinium chitralense* H. Riedl in Kew Bull. Ranunculaceae (aerial parts)	11.64 ± 0.08 µM ^a,e^	GAL 10.12 ± 0.06 µM ^a,e^ ALA 8.23 ± 0.01 µM ^a,e^	24.31 ± 0.33 µM ^a,m^	GAL 20.62 ± 0.08 µM ^a,m^ ALA 18 ± 0.06 µM ^a,m^	MCE	
Chitralinine C	*Delphinium chitralense* H. Riedl in Kew Bull. Ranunculaceae (aerial parts)	12.11 ± 0.82 µM ^a,e^	GAL 10.12 ± 0.06 µM ^a,e^ ALA 8.23 ± 0.01 µM ^a,e^	26.35 ± 0.06 µM ^a,m^	GAL 20.62 ± 0.08 µM ^a,m^ ALA 18 ± 0.06 µM ^a,m^	MCE	
Dihydropentagynine	*Delphinium denudatum* Ranunculaceae (aerial parts)	11.2 ± 0.23 µM ^a,e^	GAL 10.1 ± 0.06 µM ^a,e^	22.2 ± 0.33 µM ^a,m^	GAL 20.6 ± 0.08 µM ^a,m^	MCE	[51,68]
Isotalatizidine hydrate	*Delphinium denudatum* Ranunculaceae (aerial parts)	12.1 ± 0.43 µM ^a,e^	GAL 10.1 ± 0.06 µM ^a,e^	21.4 ± 0.23 µM ^a,m^	GAL 20.6 ± 0.08 µM ^a,m^	MCE	
Jadwarine-A	*Delphinium denudatum* Ranunculaceae (aerial parts)	9.2 ± 0.12 µM ^a,e^	GAL 10.1 ± 0.06 µM ^a,e^	19.6 ± 0.72 µM ^a,m^	GAL 20.6 ± 0.08 µM ^a,m^	MCE	
Coronaridine	*Ervatamia hainanensis* Tsiang Apocynaceae (stems)	8.6 µM ^a,e^	GAL 3.2 µM ^a,e^	nd	nd	CE	[25,48]
Voacangine	*Ervatamia hainanensis* Tsiang Apocynaceae (stems)	4.4 µM ^a,e^	GAL 3.2 µM ^a,e^	nd	nd	CE	
1-O-Acetyl-9-O-methylpseudolycorine	*Galanthus woronowii* Losinsk Amaryllidaceae (aerial parts and bulbs)	78.7 µM ^a,f^	GAL 0.15 µM ^a,f^	nd	nd	MCE	[21,48,69]
Galanthine	*Galanthus woronowii* Losinsk Amaryllidaceae (aerial parts and bulbs)	7.75 µM ^a,f^	GAL 0.15 µM ^a,f^	nd	nd	MCE	
Lycorine	*Galanthus woronowii* Losinsk Amaryllidaceae (aerial parts and bulbs)	na	GAL 0.15 µM ^a,f^	nd	nd	MCE	
Narwedine	*Galanthus woronowii* Losinsk Amaryllidaceae (aerial parts and bulbs)	11,79 µM ^a,f^	GAL 0.15 µM ^a,f^	nd	nd	MCE	
O-Methylleucotamine	*Galanthus woronowii* Losinsk Amaryllidaceae (aerial parts and bulbs)	16.42 µM ^a,f^	GAL 0.15 µM ^a,f^	nd	nd	MCE	
Salsoline	*Galanthus woronowii* Losinsk Amaryllidaceae (aerial parts and bulbs)	na	GAL 0.15 µM ^a,f^	nd	nd	MCE	
Sanguinine	*Galanthus woronowii* Losinsk Amaryllidaceae (aerial parts and bulbs)	0.007 µM ^a,f^	GAL 0.15 µM ^a,f^	nd	nd	MCE	
Sternbergine	*Galanthus woronowii* Losinsk Amaryllidaceae (aerial parts and bulbs)	0.99 µM ^a,f^	GAL 0.15 µM ^a,f^	nd	nd	MCE	
Chlidanthine	*Hieronymiella marginata* Hunz Amaryllidaceae (bulbs)	23.50 ± 0.65 µM ^a,e^	GAL 1 ± 0.05 µM ^a,e^	196.79 ± 2.67 µM ^a,m^	GAL 14 ± 0.03 µM ^a,m^	MCE	[22,48,70]
Lycorine	*Hieronymiella marginata* Hunz Amaryllidaceae (bulbs)	>200 µM ^a,e^	GAL 1 ± 0.05 µM ^a,e^	>200 µM ^a,m^	GAL 14 ± 0.03 µM ^a,m^	MCE	
Sanguinine	*Hieronymiella marginata* Hunz Amaryllidaceae (bulbs)	0.10 ± 0.03 µM ^a,e^	GAL 1 ± 0.05 µM ^a,e^	21.50 ± 0.04 µM ^a,m^	GAL 14 ± 0.03 µM ^a,m^	MCE	
Tazettine	*Hieronymiella marginata* Hunz Amaryllidaceae (bulbs)	>200 µM ^a,e^	GAL 1 ± 0.05 µM ^a,e^	>200 µM ^a,m^	GAL 14 ± 0.03 µM ^a,m^	MCE	
Hamayne	*Hippeastrum argentinum* Pax Amaryllidaceae (bulbs)	>200 µM ^a,e^	GAL 0.48 ± 0.03 µM ^a,e^	>200 µM ^a,m^	GAL 22.39 ± 0.09 µM ^a,m^	MCE	[48,69,70]
7-Hydroxyclivonine	*Hippeastrum argentinum* Pax Amaryllidaceae (bulbs)	114.07 ± 0.08 µM ^a,e^	GAL 0.48 ± 0.03 µM ^a,e^	67.3 ± 0.09 µM ^a,m^	GAL 22.39 ± 0.09 µM ^a,m^	MCE	
Lycorine	*Hippeastrum argentinum* Pax Amaryllidaceae (bulbs)	>200 µM ^a,e^	GAL 0.48 ± 0.03 µM ^a,e^	>200 µM ^a,m^	GAL 22.39 ± 0.09 µM ^a,m^	MCE	
4-O-Methylnangustine	*Hippeastrum argentinum* Pax Amaryllidaceae (bulbs)	>200 µM ^a,e^	GAL 0.48 ± 0.03 µM ^a,e^	>200 µM ^a,m^	GAL 22.39 ± 0.09 µM ^a,m^	MCE	
Montanine	*Hippeastrum argentinum* Pax Amaryllidaceae (bulbs)	>200 µM ^a,e^	GAL 0.48 ± 0.03 µM ^a,e^	>200 µM ^a,m^	GAL 22.39 ± 0.09 µM ^a,m^	MCE	
Pancracine	*Hippeastrum argentinum* Pax Amaryllidaceae (bulbs)	>200 µM ^a,e^	GAL 0.48 ± 0.03 µM ^a,e^	>200 µM ^a,m^	GAL 22.39 ± 0.09 µM ^a,m^	MCE	
Discorhabdin C	*Latrunculia biformis* Latrunculiidae (sponge)	14.5 ± 1.5 µM ^a,e^ 152 ± 12 µM ^a,f^	PHY 3.0 ± 0.3 µM ^a,e^ PHY 14.5 ± 2.0 µM ^a,f^	15.8 ± 3.5 µM ^a,m^	PHY 28.5 ± 3.0 µM ^a,m^	MCE	[48,71]
Discorhabdin G	*Latrunculia biformis* Latrunculiidae (sponge)	1.3 ± 0.2 µM ^a,e^ 116 ± 9 µM ^a,f^	PHY 3.0 ± 0.3 µM ^a,e^ PHY 14.5 ± 2.0 µM ^a,f^	7.0 ± 1.0 µM ^a,m^	PHY 28.5 ± 3.0 µM ^a,m^	MCE	
Discorhabdin B	*Latrunculia bocagei* Latrunculiidae (sponge)	5.7 ± 0.8 µM ^a,e^ 49.4 ± 7.5 µM ^a,f^	PHY 3.0 ± 0.3 µM ^a,e^ PHY 14.5 ± 2.0 µM ^a,f^	137 ± 14.5 µM ^a,m^	PHY 28.5 ± 3.0 µM ^a,m^	MCE	
Discorhabdin L	*Latrunculia bocagei* Latrunculiidae (sponge)	25.7 ± 3.0 µM ^a,e^ 158 ± 15 µM ^a,f^	PHY 3.0 ± 0.3 µM ^a,e^ PHY 14.5 ± 2.0 µM ^a,f^	531 ± 45.0 µM ^a,m^	PHY 28.5 ± 3.0 µM ^a,m^	MCE	
Lupanine	*Leontice leontopetalum* L. subsp. *ewersmannii.* Berberidaceae (tubers)	200 µg/µL—35.41 ± 3.55% ^b,k^	GAL 200 µg/µL—89.98 ± 0.61% ^b,k^	200 µg/µL—81.77 ± 2.41% ^b,n^	GAL 200 µg/µL—92.47 ± 0.63% ^b,n^	CE	[48,72]
*N*-(14-Methylallyl)-*nor*-galanthamine	*Leucojum aestivum* L. Amaryllidaceae (aerial parts)	0.16 ± 0.01 µM ^a,e^	GAL 1.82 ± 0.40 µM ^a,e^	nd	nd	MCE	[20,69]
*N*-Allyl-*nor*-galanthamine	*Leucojum aestivum* L. Amaryllidaceae (aerial parts)	0.18 ± 0.01 µM ^a,e^	GAL 1.82 ± 0.40 µM ^a,e^	nd	nd	MCE	
Casuarinine C	*Lycopodiastrum casuarinoides* Spring Lycopodiaceae (whole plant)	20.9 µM ^a,i^	HUP 0.125 µM ^a,i^	nd	nd	MCE	[48,73]
Casuarinine I	*Lycopodiastrum casuarinoides* Spring Lycopodiaceae (whole plant)	12.1 µM ^a,i^	HUP 0.125 µM ^a,i^	nd	nd	MCE	
*N*-Demethylhuperzinine	*Lycopodiastrum casuarinoides* Spring Lycopodiaceae (whole plant)	15.0 µM ^a,i^	HUP 0.125 µM ^a,i^	nd	nd	MCE	
Huperzine C	*Lycopodiastrum casuarinoides* Spring Lycopodiaceae (whole plant)	0.489 µM ^a,i^	HUP 0.125 µM ^a,i^	nd	nd	MCE	
Lycoparin C	*Lycopodium* *casuarinoides* Spring Lycopodiaceae (whole plant)	25 µM ^a,k^	nd	nd	nd	CE	[24,48]
Serratezomine D	*Lycopodium serratum* Thunb. var. *serratum* Lycopodiaceae (whole plant)	0.6 mM ^a,e^	GAL 6.4 µM ^a,e^	nd	nd	CE	[48,74]
Berberine	*Mahonia bealei* Carrière, *Mahonia fortunei* Fedde Berberidaceae (root, stem, leaf)	0.52 ± 0.06 µM ^a,k^	GAL 0.81 ± 0.08 µM ^a,k^	nd	nd	MCE	[23,48,75]
Coptisine	*Mahonia bealei* Carrière, *Mahonia fortunei* Fedde Berberidaceae (root, stem, leaf)	0.53 ± 0.04 µM ^a,k^	GAL 0.81 ± 0.08 µM ^a,k^	nd	nd	MCE	
Corypalmine	*Mahonia bealei* Carrière, *Mahonia fortunei* Fedde Berberidaceae (root, stem, leaf)	130.10 ± 9.81 µM ^a,k^	GAL 0.81 ± 0.08 µM ^a,k^	nd	nd	MCE	
Dihydroberberine	*Mahonia bealei* Carrière, *Mahonia fortunei* Fedde Berberidaceae (root, stem, leaf)	7.33 ± 0.47 µM ^a,k^	GAL 0.81 ± 0.08 µM ^a,k^	nd	nd	MCE	[23,48,75]
Epiberberine	*Mahonia bealei* Carrière, *Mahonia fortunei* Fedde Berberidaceae (root, stem, leaf)	0.80 ± 0.15 µM ^a,k^	GAL 0.81 ± 0.08 µM ^a,k^	nd	nd	MCE	
Jatrorrhizine	*Mahonia bealei* Carrière, *Mahonia fortunei* Fedde Berberidaceae (root, stem, leaf)	0.51 ± 0.04 µM ^a,k^	GAL 0.81 ± 0.08 µM ^a,k^	nd	nd	MCE	
Palmatine	*Mahonia bealei* Carrière, *Mahonia fortunei* Fedde Berberidaceae (root, stem, leaf)	0.74 ± 0.13 µM ^a,k^	GAL 0.81 ± 0.08 µM ^a,k^	nd	nd	MCE	
Stylopine	*Mahonia bealei* Carrière, *Mahonia fortunei* Fedde Berberidaceae (root, stem, leaf)	5.07 ± 0.16 µM ^a,k^	GAL 0.81 ± 0.08 µM ^a,k^	nd	nd	MCE	
Tetrahydroberberine	*Mahonia bealei* Carrière, *Mahonia fortunei* Fedde Berberidaceae (root, stem, leaf)	13.13 ± 0.4 µM ^a,k^	GAL 0.81 ± 0.08 µM ^a,k^	nd	nd	MCE	
Tetrahydropalmatine	*Mahonia bealei* Carrière, *Mahonia fortunei* Fedde Berberidaceae (root, stem, leaf)	47.56 ± 1.46 µM ^a,k^	GAL 0.81 ± 0.08 µM ^a,k^	nd	nd	MCE	
Mahanimbine	*Murraya koenigii* L. Rutaceae (leaves)	0.03 ± 0.09 mg mL^−1 a,d^	GAL 0.006 ± 0.001 mg mL^−1 a,d^	nd	nd	MCE	[48,76]
1,2-Dihydrogalanthamine	*Narcissus jonquilla* ‘Pipit’ Amaryllidaceae (bulbs)	0.19 µM ^a,e^	GAL 0.27 µM ^a,e^	nd	nd	BTLC by Mroczek	[77]
Haemanthamine	*Narcissus poeticus* ‘Pink Parasol’ Amaryllidaceae (bulbs)	>500 µM ^a,f^	GAL 1.7 ± 0.1 µM ^a,f^ HUP 0.033 ± 0.001 µM ^a,f^	>500 µM ^a,l^	GAL 42.3 ± 1.3 µM ^a,l^ HUP >500 µM ^a,l^	MCE	[48,78]
Hippeastrine	*Narcissus poeticus* ‘Pink Parasol*’* Amaryllidaceae (bulbs)	>500 µM ^a,f^	GAL 1.7 ± 0.1 µM ^a,f^ HUP 0.033 ± 0.001 µM ^a,f^	>500 µM ^a,l^	GAL 42.3 ± 1.3 µM ^a,l^ HUP >500 µM ^a,l^	MCE	
Homolycorine	*Narcissus poeticus* ‘Pink Parasol*’* Amaryllidaceae (bulbs)	64 ± 4 µM ^a,f^	GAL 1.7 ± 0.1µM ^a,f^ HUP 0.033 ± 0.001µM ^a,f^	151 ± 19 µM ^a,l^	GAL 42.3 ± 1.3 µM ^a,l^ HUP >500 µM ^a,l^	MCE	
Incartine	*Narcissus poeticus* ‘Pink Parasol*’* Amaryllidaceae (bulbs)	208 ± 14 µM ^a,f^	GAL 1.7 ± 0.1µM ^a,f^ HUP 0.033 ± 0.001 µM ^a,f^	>500 µM ^a,l^	GAL 42.3 ± 1.3 µM ^a,l^ HUP >500 µM ^a,l^	MCE	
Lycoramine	*Narcissus poeticus* ‘Pink Parasol*’* Amaryllidaceae (bulbs)	456 ± 57 µM ^a,f^	GAL 1.7 ± 0.1 µM ^a,f^ HUP 0.033 ± 0.001 µM ^a,f^	>500 µM ^a,l^	GAL 42.3 ± 1.3 µM ^a,l^ HUP >500 µM ^a,l^	MCE	
Masonine	*Narcissus poeticus* ‘Pink Parasol*’* Amaryllidaceae (bulbs)	304 ± 34 µM ^a,f^	GAL 1.7 ± 0.1 µM ^a,f^ HUP 0.033 ± 0.001 µM ^a,f^	229 ± 24 µM ^a,l^	GAL 42.3 ± 1.3 µM ^a,l^ HUP >500 µM ^a,l^	MCE	
Narcipavline	*Narcissus poeticus* ‘Pink Parasol*’* Amaryllidaceae (bulbs)	208 ± 37 µM ^a,f^	GAL 1.7 ± 0.1 µM ^a,f^ HUP 0.033 ± 0.001 µM ^a,f^	24.4 ± 1.2 µM ^a,l^	GAL 42.3 ± 1.3 µM ^a,l^ HUP >500 µM ^a,l^	MCE	
Narwedine	*Narcissus poeticus* ‘Pink Parasol*’* Amaryllidaceae (bulbs)	281 ± 33 µM ^a,f^	GAL 1.7 ± 0.1 µM ^a,f^ HUP 0.033 ± 0.001 µM ^a,f^	>500 µM ^a,l^	GAL 42.3 ± 1.3 µM ^a,l^ HUP >500 µM ^a,l^	MCE	
*nor*-Lycoramine	*Narcissus poeticus* ‘Pink Parasol*’* Amaryllidaceae (bulbs)	>500 µM ^a,f^	GAL 1.7 ± 0.1 µM ^a,f^ HUP 0.033 ± 0.001 µM ^a,f^	>500 µM ^a,l^	GAL 42.3 ± 1.3 µM ^a,l^ HUP >500 µM ^a,l^	MCE	
Oduline	*Narcissus poeticus* ‘Pink Parasol*’* Amaryllidaceae (bulbs)	>500 µM ^a,f^	GAL 1.7 ± 0.1 µM ^a,f^ HUP 0.033 ± 0.001 µM ^a,f^	>500 µM ^a,l^	GAL 42.3 ± 1.3 µM ^a,l^ HUP >500 µM ^a,l^	MCE	
*seco*-Isopowellaminone	*Narcissus poeticus* ‘Pink Parasol*’* Amaryllidaceae (bulbs)	293 ± 33 µM ^a,f^	GAL 1.7 ± 0.1 µM ^a,f^ HUP 0.033 ± 0.001 µM ^a,f^	>500 µM ^a,l^	GAL 42.3 ± 1.3 µM ^a,l^ HUP >500 µM ^a,l^	MCE	
Incartine	*Narcissus jonquila* var. *henriquesii* Samp. Amaryllidaceae (bulbs)	208.2 ± 14.3 µM ^a,f^	GAL 1.7 ± 0.06 µM ^a,f^ HUP 0.03 ± 0.0 µM ^a,f^ PHY 0.06 ± 0.0 µM ^a,f^	943.4 ± 140.7 µM ^a,l^	GAL 42.3 ± 1.3 µM ^a,l^ HUP >1000 µM ^a,l^ PHY 0.13 ± 0.0 µM ^a,l^	MCE	[48,79]
Narwedine	*Narcissus poeticus* ’Brackenhurst’ Amaryllidaceae (bulbs)	281.2 ± 33.9 µM ^a,f^	GAL 1.7 ± 0.06 µM ^a,f^ HUP 0.03 ± 0.0 µM ^a,f^ PHY 0.06 ± 0.0 µM ^a,f^	911.3 ± 68.7 µM ^a,l^	GAL 42.3 ± 1.3 µM ^a,l^ HUP >1000 µM ^a,l^ PHY 0.13 ± 0.0 µM ^a,l^	MCE	
11-Hydroxygalanthine	*Narcissus tazetta* subsp. *tazetta* L Amaryllidaceae (bulbs and leaves)	0.67 µM ^a,e^	GAL 0.15 µM ^a,e^	18.17 µM ^a,m^	GAL 2.47µM ^a,m^	MCE	[48,80]
9-*O*-Demetil-2-α-hydroxyhomolycorine	*Narcissus tazetta* subsp. *tazetta* L Amaryllidaceae (bulbs and leaves)	19.84 µM ^a,e^	GAL 0.15 µM ^a,e^	na	GAL 2.47 µM ^a,m^	MCE	
Narcissidine	*Narcissus tazetta* subsp. *tazetta* L Amaryllidaceae (bulbs and leaves)	1.85 µM ^a,e^	GAL 0.15 µM ^a,e^	na	GAL 2.47 µM ^a,m^	MCE	
Pancratinine-C	*Narcissus tazetta* subsp. *tazetta* L Amaryllidaceae (bulbs and leaves)	na	GAL 0.15 µM ^a,e^	32.04 µM ^a,m^	GAL 2.47 µM ^a,m^	MCE	
Pseudolycorine	*Narcissus tazetta* subsp. *tazetta* L Amaryllidaceae (bulbs and leaves)	32.51 µM ^a,e^	GAL 0.15 µM ^a,e^	21.64 µM ^a,m^	GAL 2.47 µM ^a,m^	MCE	
Angustidine	*Nauclea officinalis* Merr. & Chun. Rubiaceae (bark)	21.72 µM ^a,e^	GAL 0.94 µM ^a,e^	1.03 µM ^a,m^	GAL 28.29 µM ^a,m^	CE	[19,48,81]
Angustine	*Nauclea officinalis* Merr. & Chun. Rubiaceae (bark)	100 μg mL^−1^—40.19 ± 0.65% ^b,e^	GAL 0.94 µM ^a,e^	4.98 µM ^a,m^	GAL 28.29 µM ^a,m^	CE	
Angustoline	*Nauclea officinalis* Merr. & Chun. Rubiaceae (bark)	261.89 µM ^a,e^	GAL 0.94 µM ^a,e^	25.10 µM ^a,m^	GAL 28.29 µM ^a,m^	CE	
Harmane	*Nauclea officinalis* Merr. & Chun. Rubiaceae (bark)	300.68 µM ^a,e^	GAL 0.94 µM ^a,e^	13.18 µM ^a,m^	GAL 28.29 µM ^a,m^	CE	
Nauclefine	*Nauclea officinalis* Merr. & Chun. Rubiaceae (bark)	100 μg mL^−1^—34.61 ± 4.84% ^b,e^	GAL 0.94 µM ^a,e^	7.70 µM ^a,m^	GAL 28.29 µM ^a,m^	CE	
7,8,13,14-Dehydroorientalidine	*Papaver setiferum* Goldblatt Papaveraceae (capsules)	nd	NEO 6.0 ± 1.1 µM ^a,e^	nd	NEO 92.7 ± 2.2 µM ^a,m^	MCE	[48,82,83]
7,8-Didehydromecambridine TFA salt	*Papaver setiferum* Goldblatt Papaveraceae (capsules)	10.3 ± 1.1 µM ^a,e^	NEO 6.0 ± 1.1 µM ^a,e^	100 ± 5 µM ^a,m^	NEO 92.7 ± 2.2 µM ^a,m^	MCE	
7,8- Didehydroorientalidine TFA salt	*Papaver setiferum* Goldblatt Papaveraceae (capsules)	3.4 ± 4.7 µM ^a,e^	NEO 6.0 ± 1.1 µM ^a,e^	98.5 ± 0.6 µM ^a,m^	NEO 92.7 ± 2.2 µM ^a,m^	MCE	
Alborine	*Papaver setiferum* Goldblatt Papaveraceae (capsules)	6.8 ± 4.5 µM ^a,e^	NEO 6.0 ± 1.1 µM ^a,e^	63.1 ± 0.5 µM ^a,m^	NEO 92.7 ± 2.2 µM ^a,m^	MCE	
Isothebaine	*Papaver setiferum* Goldblatt Papaveraceae (capsules)	260 ± 1 µM ^a,e^	NEO 6.0 ± 1.1 µM ^a,e^	2.8 ± 3.0 µM ^a,m^	NEO 92.7 ± 2.2 µM ^a,m^	MCE	
*N*-Methylcodamine	*Papaver setiferum* Goldblatt Papaveraceae (capsules)	nd	NEO 6.0 ± 1.1 µM ^a,e^	221 ± 1 µM ^a,m^	NEO 92.7 ± 2.2 µM ^a,m^	MCE	
*N*-Methylisothebainium	*Papaver setiferum* Goldblatt Papaveraceae (capsules)	nd	NEO 6.0 ± 1.1 µM ^a,e^	7.1 ± 2.7 µM ^a,m^	NEO 92.7 ± 2.2 µM ^a,m^	MCE	
*N*-Methylorientaline	*Papaver setiferum* Goldblatt Papaveraceae (capsules)	nd	NEO 6.0 ± 1.1 µM ^a,e^	342 ± 3 µM ^a,m^	NEO 92.7 ± 2.2 µM ^a,m^	MCE	
Orientalidine	*Papaver setiferum* Goldblatt Papaveraceae (capsules)	5.0 ± 1.0 µM ^a,e^	NEO 6.0 ± 1.1 µM ^a,e^	104 ± 4 µM ^a,m^	NEO 92.7 ± 2.2 µM ^a,m^	MCE	
Salutaridine	*Papaver setiferum* Goldblatt Papaveraceae (capsules)	nd	NEO 6.0 ± 1.1 µM ^a,e^	335 ± 4 µM ^a,m^	NEO 92.7 ± 2.2 µM ^a,m^	MCE	
19(*S*)-Hydroxyibogamine	*Tabernaemontana bufalina* Lour. (Apocynaceae)	nd	nd	20.1 µM ^a,m^	TAC 0.025 µM ^a,m^	MCE	[48,84,85]
3α-Dihydrocadambine	*Uncaria rhynchophylla* Miq. ex Havil Rubiaceae (stems)	37.01 *±* 1.57 µM ^a,e^	TAC 4.39 *±* 0.80 µM ^a,e^	33.34 ± 0.51 µM ^a,m^	TAC 3.25 *±* 1.86 µM ^a,m^	MCE	[48,86]
7-*epi*-Javaniside	*Uncaria rhynchophylla* Miq. ex Havil Rubiaceae (stems)	2.85 ± 0.50 µM ^a,e^	TAC 4.39 *±* 0.80 µM ^a,e^	2.13 ± 0.10 µM ^a,m^	TAC 3.25 *±* 1.86 µM ^a,m^	MCE	
Cadambine	*Uncaria rhynchophylla* Miq. ex Havil Rubiaceae (stems)	26.12 ± 2.12 µM ^a,e^	TAC 4.39 *±* 0.80 µM ^a,e^	30.69 ± 0.69 µM ^a,m^	TAC 3.25 *±* 1.86 µM ^a,m^	MCE	
Strictosamide	*Uncaria rhynchophylla* Miq. ex Havil Rubiaceae (stems)	46.57 ± 0.58µM ^a,e^	TAC 4.39 *±* 0.80 µM ^a,e^	6.47 ± 0.72 µM ^a,m^	TAC 3.25 *±* 1.86 µM ^a,m^	MCE	
Vincosamide	*Uncaria rhynchophylla* Miq. ex Havil Rubiaceae (stems)	12.4 *±* 0.86 µM ^a,e^	TAC 4.39 *±* 0.80 µM ^a,e^	23.18 *±* 0.14 µM ^a,m^	TAC 3.25 *±* 1.86 µM ^a,m^	MCE	
Deoxyvobtusine lactone	*Voacanga globosa* Merr. Apocynaceae (leaves)	10^−4.3^ M—91% ^b,e^	GAL 0.64 µM ^a,e^	20.2 µM ^a,m^	GAL 8.40 µM ^a,m^	MCE	[87,88,89]
Deoxyvobtusine	*Voacanga globosa* Merr. Apocynaceae (leaves)	10^−4.3^ M—87% ^b,e^	GAL 0.64 µM ^a,e^	6.2 µM ^a,m^	GAL 8.40 µM ^a,m^	MCE	
Globospiramine	*Voacanga globosa* Merr. Apocynaceae (leaves)	10^−4.3^ M—94% ^b,e^	GAL 0.64 µM ^a,e^	16.4 µM ^a,m^	GAL 8.40 µM ^a,m^	MCE	
Vobtusine lactone	*Voacanga globosa* Merr. Apocynaceae (leaves)	10^−4.3^ M—90% ^b,e^	GAL 0.64 µM ^a,e^	18.0 µM ^a,m^	GAL 8.40 µM ^a,m^	MCE	
ANTHRANOIDS							
2-Geranylemodin	*Psorospermum glaberrimum* Hochr. Hypericaceae (stem bark)	0.1 mM—12.9% ^b,e^	GAL 0.50 ± 0.001 µM ^a,e^	11.30 ± 0.23 µM ^a,m^	GAL 8.50 ± 0.001 µM ^a,m^	MCE	[48,90]
3-Prenyloxyemodin	*Psorospermum glaberrimum* Hochr. Hypericaceae (stem bark)	0.1 mM—35.0% ^b,e^	GAL 0.50 ± 0.001 µM ^a,e^	13.3 ± 1.10 µM ^a,m^	GAL 8.50 ± 0.001 µM ^a,m^	MCE	
Acetylvismione D	*Psorospermum glaberrimum* Hochr. Hypericaceae (stem bark)	0.1 mM—45.70% ^b,e^	GAL 0.50 ± 0.001 µM ^a,e e^	10.1 ± 0.20 µM ^a,m^	GAL 8.50 ± 0.001 µM ^a,m^	MCE	
Bianthrone 1a	*Psorospermum glaberrimum* Hochr. Hypericaceae (stem bark)	63.0 ± 0.46 µM ^a,e^	GAL 0.50 ± 0.001 µM ^a,e a,e^	9.25 ± 0.25 µM ^a,m^	GAL 8.50 ± 0.001 µM ^a,m^	MCE	
3-Geranyloxyemodin anthrone	*Psorospermum glaberrimum* Hochr. Hypericaceae (stem bark)	100 µM—5.4% ^b,e^	GAL 0.50 ± 0.001 µM ^a,e e^	11.60 ± 0,20 µM ^a,m^	GAL 8.50 ± 0.001 µM ^a,m^	MCE	
3-Prenyloxyemodin anthrone	*Psorospermum glaberrimum* Hochr. Hypericaceae (stem bark)	100 µM—13.8% ^b,e^	GAL 0.50 ± 0.001 µM ^a,e^	10.1 ± 0.5 µM ^a,m^	GAL 8.50 ± 0.001 µM ^a,m^	MCE	
Emodin	*Talaromyces aurantiacus* FL 15 (strain from leave *Huperzia serrata*)	>100 µM ^a,e^	RIV 1.82 ± 0.13 µM ^a,e^ HUP 0.045 ± 0.01 µM ^a,e^	>100 µM ^a,m^	nd	MCE	[48,91,92]
Physcion	*Talaromyces aurantiacus* FL 15 (strain from leave *Huperzia serrata*)	>100 µM ^a,e^	RIV 1.82 ± 0.13 µM ^a,e^ HUP 0.045 ± 0.01 µM ^a,e^	>100 µM ^a,m^	nd	MCE	
Chrysophanol	*Talaromyces aurantiacus* FL 15 (strain from leave *Huperzia serrata*)	>100 µM ^a,e^	RIV 1.82 ± 0.13 µM ^a,e^ HUP 0.045 ± 0.01 µM ^a,e,^	>100 µM ^a,m^	nd	MCE	
BIBENZYLS							
3,3′-Dihydroxy-4-(4-hydroxybenzyl)-5-methoxybibenzyl	*Bletilla striata* Reichb. f. Orchidaceae (tuber)	25 μg mL^−1^—2.6 ± 2.8% ^b,e^	GAL 25 μg mL^−1^—94.8 ± 0.9% ^b,e^	25 μg mL^−1^—22.6 ± 2.1% ^b,m^	GAL 25 μg mL^−1^—64.2 ± 0.6% ^b,m^ 46.3 ± 5.8 µM ^a,m^ TAC 0.0101 ± 0.0005 µM ^a,m^	MCE	[37,48]
3′,5-Dihydroxy-2-(4-hydroxybenzyl)-3-methoxybibenzyl	*Bletilla striata* Reichb. f. Orchidaceae (tuber)	25 μg mL^−1^—5.0 ± 1.5% ^b,e^	GAL 25 μg mL^−1^—94.8 ± 0.9% ^b,e^	25 μg mL^−1^—51.3 ± 2.0% ^b,m^ 80.3 ± 5.2 µM ^a,m^	GAL 25 μg mL^−1^—64.2 ± 0.6% ^b,m^ 46.3 ± 5.8 µM ^a,m^ TAC 0.0101 ± 0.0005 µM ^a,m^	MCE	
Bulbocol	*Bletilla striata* Reichb. f. Orchidaceae (tuber)	25 μg mL^−1^—16.3 ± 3.8% ^b,e^	GAL 25 μg mL^−1^—94.8 ± 0.9% ^b,e^	25 μg mL^−1^—67.7 ± 0.3% ^b,m^ 33.5 ± 3.7 µM ^a,m^	GAL 25 μg mL^−1^—64.2 ± 0.6% ^b,m^ 46.3 ± 5.8 µM ^a,m^ TAC 0.0101 ± 0.0005 µM ^a,m^	MCE	
Gymconopin D	*Bletilla striata* Reichb. f. Orchidaceae (tuber)	25 μg mL^−1^—48.1 ± 6.3% ^b,e^	GAL 25 μg mL^−1^—94.8 ± 0.9% ^b,e^	25 μg mL^−1^—66.2 ± 3.4% ^b,m^ 40.5 ± 5.6 µM ^a,m^	GAL 25 μg mL^−1^—64.2 ± 0.6% ^b,m^ 46.3 ± 5.8 µM ^a,m^ TAC 0.0101 ± 0.0005 µM ^a,m^	MCE	
COUMARINS							
Scopoletin	*Scopolia carniolica* Jaqc. Solanaceae (roots)	168.6 µM ^a,e^	GAL 3.2 µM ^a,e^	nd	nd	MCE	[16,48,93,94,95]
Decursinol	*Angelica gigas* Nakai Apiaceae (underground parts)	28 μM ^a,k^	nd	nd	nd	MCE	[48,96,97,98]
Isoimperatorin	*Angelica gigas* Nakai Apiaceae (underground parts)	69 μM ^a,k^	nd	nd	nd	MCE	
Marmesin	*Angelica gigas* Nakai Apiaceae (underground parts)	67 μM ^a,k^	nd	nd	nd	MCE	
Nodakenin	*Angelica gigas* Nakai Apiaceae (underground parts)	68 μM ^a,k^	nd	nd	nd	MCE	
Xanthotoxin	*Angelica gigas* Nakai Apiaceae (underground parts)	54 μM ^a,k^	nd	nd	nd	MCE	
Bergapten	*Angelica officinalis* L. Apiaceae (fruits)	25 µg mL^−1^— 32.65 ± 6.10% ^b,e^ 100 µg mL^−1^—nd	GAL 100 µg mL^−1^—98.97 ± 0.24% ^b,e^	25 µg mL^−1^—86.69 ± 2.56% ^b,m^ 100 µg mL^−1^- nd	GAL 100 µg mL^−1^—80.31 ± 1.14% ^b,m^	MCE	[48,99,100]
Imperatorin	*Angelica officinalis* L. Apiaceae (fruits)	25 µg mL^−1^— 18.76 ± 1.07% ^b,e^ 100 µg mL^−1^—46.11 ± 0.92% ^b,e^	GAL 100 µg mL^−1^—98.97 ± 0.24% ^b,e^	25 µg mL^−1^— 37.46 ± 1.09% ^b,m^ 100 µg mL^−1^— 83.98 ± 0.99% ^b,m^	GAL 100 µg mL^−1^—80.31 ± 1.14% ^b,m^	MCE	
Xanthotoxin	*Angelica officinalis* L. Apiaceae (fruits)	25 µg mL^−1^— 38.23 ± 0.06% ^b,e^ 100 µg mL^−1^—66.08 ± 2.88% ^b,e^	GAL 100 µg mL^−1^—98.97 ± 0.24% ^b,e^	25 µg mL^−1^—63.60 ± 1.78% ^b,m^ 100 µg mL^−1^—88.04 ± 0.83% ^b,m^	GAL 100 µg mL^−1^—80.31 ± 1.14% ^b,m^	MCE	
Heraclenol-2′-O-angelate	*Archangelicae officinalis* L. Apiaceae (roots)	>1000 μM ^a,e^	GAL 0.37 ± 1.1 μM ^a,e^	7.5 ± 1.8 μM ^a,m^	GAL 8.3 ± 2.6 μM ^a,m^	BTLC by Marston et al. (2002)	[28,48,101]
Imperatorin	*Archangelicae officinalis* L. Apiaceae (fruits)	156 ± 15 μM ^a,e^	GAL 0.37 ± 1.1 μM ^a,e^	14.4 ± 3.2 μM ^a,m^	GAL 8.3 ± 2.6 μM ^a,m^	BTLC by Marston et al. (2002)	
Isoimperatorin	*Citrus hystrix* DC. Rutaceae (peels of fruits)	nd	nd	23 ± 0.2 µM ^a,m^	GAL 3.2 ± 0.2 µM ^a,m^	MCE	[27,48]
6′,7′- Dihydroxybergamottin	*Citrus hystrix* DC Rutaceae (peels of fruits)	nd	nd	15.4 ± 0.3 µM ^a,m^	GAL 3.2 ± 0.2 µM ^a,m^	MCE	
6′-Hydroxy-7′-methoxybergamottin	*Citrus hystrix* DC. Rutaceae (peels of fruits)	nd	nd	11.2 ± 0.1 µM ^a,m^	GAL 3.2 ± 0.2 µM ^a,m^	MCE	
5,7-Dihydroxy-8-(3-methylbutanoyl)- 6-[(*E*)-3,7-dimethylocta-2,6-dienyl]-4-phenyl- 2H-chromen-2-one	*Mesua elegans* Kosterm. Clusiaceae (bark)	3.06 ± 0.04 µM ^a,e^	TAC 0.074 ± 0.012 µM ^a,e^	nd	nd	CE	[29,48]
Mesuagenin A	*Mesua elegans* Kosterm. Clusiaceae (bark)	1.06 ± 0.04 µM ^a,e^	TAC 0.074 ± 0.012 µM ^a,e^	nd	nd	CE	
Mesuagenin B	*Mesua elegans* Kosterm. Clusiaceae (bark)	0.70 ± 0.10 µM ^a,e^	TAC 0.074 ± 0.012 µM ^a,e^	nd	nd	CE	
Mesuagenin D	*Mesua elegans* Kosterm. Clusiaceae (bark)	8.73 ± 0.25 µM ^a,e^	TAC 0.074 ± 0.012 µM ^a,e^	nd	nd	CE	
Lucidafuranocoumarin A	*Peucedanum alsaticum* L. Apiaceae (fruits)	na	GAL 100 µg mL^−1^—92.14 ± 2.49% ^b,k^ 1.82 ± 0.22 µg mL^−1 a,k^	100 µg mL^−1^—40.66 ± 1.25% ^b,n^	GAL 100 µg mL^−1^—81.93 ± 2.52% ^b,n^ 22.16 ± 0.91 µg mL^−1 a,n^	MCE	[102]
Bergamottin	*Peucedanum alsaticum* L. Apiaceae (fruits)	100 µg mL^−1^—4.00 ± 0.82% ^b^	GAL 100 µg mL^−1^—92.14 ± 2.49% ^b,k^ 1.82 ± 0.22 µg mL^−1 a,k^	100 µg mL^−1^—17.65 ± 1.50% ^b^	GAL 100 µg mL^−1^—81.93 ± 2.52% ^b,n^ 22.16 ± 0.91 µg mL^−1 a,n^	MCE	
CHROMONES							
Sargachromanol G	*Sargassum siliquastrum* Sargassaceae (strains)	1.81 ± 0.020 µM ^a,e^	BER 1.01 ± 0.01 µM ^a,e^ TAC 0.22 ± 0.004 µM ^a,e^	10.79 ± 0.65 µM ^a,m^	TAC 0.014 ± 0.0043 µM ^a,m^	MCE	[48,59,60]
Sargachromanol I	*Sargassum siliquastrum* Sargassaceae (strains)	0.79 ± 0.07 µM ^a,e^	BER 1.01 ± 0.01 µM ^a,e^ TAC 0.22 ± 0.004 µM ^a,e^	13.69 ± 5.07 µM ^a,m^	TAC 0.014 ± 0.0043 µM ^a,m^	MCE	
DIARYLHEPTANOIDS							
(−)-Alpininoid B	*Alpinia officinarum* Hance Zingiberaceae (rhizomes)	100 µM—87.6 ± 0.1% ^b,e^ 2.6 ± 4.2 µM ^a,e^	TAC 111.8 ± 4.6 µM ^a,e^	100 µM—64.7 ± 1.4% ^b,m^ 35.2 ± 0.7 µM ^a,m^	TAC 8.9 ± 2.4 µM ^a,m^	MCE	[31,66]
(4*E*)^−1^,7-Diphenyl-4-hepten-3-one	*Alpinia officinarum* Hance Zingiberaceae (rhizomes)	100 µM—98.0 ± 0.9% ^b,e^ 23.9 ± 2.6 µM ^a,e^	TAC 111.8 ± 4.6 µM ^a,e^	100 µM—62.3 ± 3.5% ^b,m^ 70.7 ± 2.5 µM ^a,m^	TAC 8.9 ± 2.4 µM ^a,m^	MCE	
Dihydroyashsbushiketol	*Alpinia officinarum* Hance Zingiberaceae (rhizomes)	100 µM—36.2 ± 1.9% ^b,e^	TAC 111.8 ± 4.6 µM ^a,e^	100 µM—15.7 ± 2.1% ^b,m^	TAC 8.9 ± 2.4 µM ^a,m^	MCE	
(4*E*)-7-(4-Hydroxyphenyl)-1-phenyl-4-hepten-3-one	*Alpinia officinarum* Hance Zingiberaceae (rhizomes)	100 µM –57.9 ± 3.2% ^b,e^ 87.3 ± 3.4 µM ^a,e^	TAC 111.8 ± 4.6 µM ^a,e^	100 µM—41.1 ± 0.1% ^b,m^	TAC 8.9 ± 2.4 µM ^a,m^	MCE	
(4*E*)-7-(4-Hydroxy-3-methoxyphenyl)-1-phenyl-hept-4-en-3-one	*Alpinia officinarum* Hance Zingiberaceae (rhizomes)	100 µM—76.6 ± 0.3% ^b,e^ 39.1 ± 2.3 µM ^a,e^	TAC 111.8 ± 4.6 µM ^a,e^	100 µM—43.7 ± 1.4% ^b,m^	TAC 8.9 ± 2.4 µM ^a,m^	MCE	
(5*R*)-7-(4-Hydroxy-3-methoxyphenyl)-5-methoxy-1-phenyl-3-heptanone	*Alpinia officinarum* Hance Zingiberaceae (rhizomes)	100 µM—35.3 ± 1.0% ^b,e^	TAC 111.8 ± 4.6 µM ^a,e^	100 µM—21.5 ± 0.6% ^b,m^	TAC 8.9 ± 2.4 µM ^a,m^	MCE	
Kaempferide	*Alpinia officinarum* Hance Zingiberaceae (rhizomes)	100 µM—67.2 ± 1.8% ^b,e^ 31.9 ± 2.0 µM ^a,e^	TAC 111.8 ± 4.6 µM ^a,e^	100 µM –47.6 ± 1.6% ^b,m^	TAC 8.9 ± 2.4 µM ^a,m^	MCE	
Galangin	*Alpinia officinarum* Hance Zingiberaceae (rhizomes)	100 µM—65.4 ± 4.5% ^b,e^ 70.1 ± 1.5 µM ^a,e^	TAC 111.8 ± 4.6 µM ^a,e^	100 µM—63.6 ± 3.1% ^b,m^ 61.4 ± 1.4 µM ^a,m^	TAC 8.9 ± 2.4 µM ^a,m^	MCE	
DITERPENES							
Dihydrotanshinone	*Salvia miltiorhiza* Bunge Lamiaceae (roots)	1 μM ^a,d^	PHY 0.25 µM ^a,d^	nd	nd	MCE	[38,103]
Cryptotanshinone	*Salvia miltiorhiza* Bunge Lamiaceae (roots)	7 μM ^a,d^	PHY 0.25 µM ^a,d^	nd	nd	MCE	
Tanshinone I	*Salvia miltiorhiza* Bunge Lamiaceae (roots)	>50 μM ^a,d^	PHY 0.25 µM ^a,d^	nd	nd	MCE	
Tanshionone IIA	*Salvia miltiorhiza* Bunge Lamiaceae (roots)	>140 μM ^a,d^	PHY 0.25 µM ^a,d^	nd	nd	MCE	
Scapaundulin C	*Scapania undulate* L. Scapaniaceae	>250 ng ^c,e^	GAL >10 ng ^c,e^	nd	nd	BTLC by Marston et al. (2002)	[104,105]
Scapaundulin A	*Scapania undulate* L. Scapaniaceae	>250 ng ^c,e^	GAL >10 ng ^c,e^	nd	nd	BTLC by Marston et al. (2002)	
5α, 8α, 9α-Trihydroxy-13*E*-labden-12-one	*Scapania undulate* L. Scapaniaceae	>250 ng ^c,e^	GAL >10 ng ^c,e^	nd	nd	BTLC by Marston et al. (2002)	
5α, 8α- Dihydroxy-13*E*-labden-12-one	*Scapania undulate* L. Scapaniaceae	>250 ng ^c,e^	GAL >10 ng ^c,e^	nd	nd	BTLC by Marston et al. (2002)	
(13*S*)-15-Hydroxylabd-8 (17)-en-19-oic acid	*Scapania undulate* L. Scapaniaceae	>500 ng ^c,e^	GAL >10 ng ^c,e^	nd	nd	BTLC by Marston et al. (2002)	
FATTY ACID							
(2*E*,4*E*,6R)-6-Hydroxydeca- 2,4-dienoic acid.	*Lycopodiella cernua* L. Lycopodiaceae (whole plants)	0.22 ± 0.03 µM ^a,k^	BER 0.10 ± 0.01 µM ^a,k^	>30 µM ^a,n^	BER 1.09 ± 0.17 µM ^a,n^	MCE	[48,106]
FLAVONOIDS							
3-Methoxy quercetin	*Agrimonia pilosa* Ledeb*.* Rosaceae (leaves)	37.9 μM ^a,e^	DEH 37.8 μM ^a,e^	nd	nd	MCE	[48,107]
Quercetin	*Agrimonia pilosa* Ledeb*.* Rosaceae (leaves)	19.8 μM ^a,e^	DEH 37.8 μM ^a,e^	nd	nd	MCE	
Quercitrin	*Agrimonia pilosa* Ledeb*.* Rosaceae (leaves)	66.9 μM ^a,e^	DEH 37.8 μM ^a,e^	nd	nd	MCE	
Tiliroside	*Agrimonia pilosa* Ledeb*.* Rosaceae (leaves)	23.5 μM ^a,e^	DEH 37.8 μM ^a,e^	nd	nd	MCE	
Linarin	*Buddleja davidii* Franch. Buddlejaceae (leaves)	>10 ng ^c,e^	HUP >1 ng ^c,e^	nd	nd	BTLC by Marston et al. (2002)	[101,104]
Garcineflavonol A	*Garcinia atroviridis* Griff. ex T. Anderson Clusiaceae (stem bark)	100 μg mL^−1^—68.45 ± 0.97% ^b,e^ 14.04 ± 0.77 μg mL^−1 a,e^	PHY 0.05 ± 0.01 μg mL^−1 a,e^	14.50 ± 0.47 μg mL^−1 a,m^	PHY 0.14 ± 0.015 μg mL^−1 a,m^	MCE	[48,108,109]
Quercetin	*Ginkgo biloba* L. Ginkgoaceae (leaves)	95.8 μg mL^−1 a,h^	CHL 12.4 μg mL^−1 a,h^	nd	nd	MCE	[48,110,111]
Quercetin- 3-O-𝛼-L-rhamnopyranosyl- (1 → 6)-𝛽-D-glucopyranoside	*Ginkgo biloba* L. Ginkgoaceae (leaves)	73.1 μg mL^−1 a,h^	CHL 12.4 μg mL^−1 a,h^	nd	nd	MCE	
Quercetin-3-O- 𝛽-D-glucopyranoside	*Ginkgo biloba* L. Ginkgoaceae (leaves)	57.8 μg mL^−1 a,h^	CHL 12.4 μg mL^−1 a,h^	nd	nd	MCE	
Quercetin-3-O-𝛼-L-rhamnopyranoside	*Ginkgo biloba* L. Ginkgoaceae (leaves)	110.9 μg mL^−1 a,h^	CHL 12.4 μg mL^−1 a,h^	nd	nd	MCE	
Quercetin-3-O-𝛼-L-rhamnopyranosyl- (1 → 4)-O-𝛼-L-rhamnopyranosyl- (1 → 2)-𝛽-D-glucopyranoside	*Ginkgo biloba* L. Ginkgoaceae (leaves)	112.6 μg mL^−1 a,h^	CHL 12.4 μg mL^−1 a,h^	nd	nd	MCE	
Taxifolin	*Ginkgo biloba* L. Ginkgoaceae (leaves)	133.1 μg mL^−1 a,h^	CHL 12.4 μg mL^−1 a,h^	nd	nd	MCE	
Quercetin-3-O-neohesperidoside	*Lysimachia clethroides* Duby Primulaceae (whole plant)	6.98 ± 0.47 µM ^a,e^	BER 1.01 ± 0.01 µM ^a,e^ TAC 0.22 ± 0.004 µM ^a,e^	>40 µM ^a,m^	TAC 0.014 ± 0.0043 µM ^a,m^	MCE	[48,59,60]
Diplacone	*Paulownia* *tomentosa* Steud. Paulowniaceae (fruits)	7.2 ± 0.6 µM ^a,f^	PHY 0.15 ± 0.03 µM ^a,f^	1.4 ± 0.3 µM ^a,m^	PHY 3.7 ± 0.6 µM ^a,m^	MCEF	[34,48,112]
3′-O-Methyldiplacol	*Paulownia* *tomentosa* Steud. Paulowniaceae (fruits)	48.5 ± 2.1 µM ^a,f^	PHY 0.15 ± 0.03 µM ^a,f^	11.2 ± 2.1 µM ^a,m^	PHY 3.7 ± 0.6 µM ^a,m^	MCEF	
3′-O-Methyldiplacone	*Paulownia* *tomentosa* Steud. Paulowniaceae (fruits)	109.2 ±8.4 µM ^a,f^	PHY 0.15 ± 0.03 µM ^a,f^	24.5 ± 1.2 µM ^a,m^	PHY 3.7 ± 0.6 µM ^a,m^	MCEF	
4′-O-Methyldiplacone	*Paulownia* *tomentosa* Steud. Paulowniaceae (fruits)	92.4 ± 4.1 µM ^a,f^	PHY 0.15 ± 0.03 µM ^a,f^	25.6 ± 1.6 µM ^a,m^	PHY 3.7 ± 0.6 µM ^a,m^	MCEF	
4′-O-Methyldiplacol	*Paulownia* *tomentosa* Steud. Paulowniaceae (fruits)	31.9 ± 1.2 µM ^a,f^	PHY 0.15 ± 0.03 µM ^a,f^	12.7 ± 1.3 µM ^a,m^	PHY 3.7 ± 0.6 µM ^a,m^	MCEF	
6-Geranyl-3,3′,5,5′,7-pentahydroxy- 4′-methoxyflavane	*Paulownia* *tomentosa* Steud. Paulowniaceae (fruits)	15.6 ± 0.8 µM ^a,f^	PHY 0.15 ± 0.03 µM ^a,f^	3.8 ± 0.8 µM ^a,m^	PHY 3.7 ± 0.6 µM ^a,m^	MCEF	
6-Geranyl-3′,5,5′,7-tetrahydroxy- 4′-methoxyflavanone	*Paulownia* *tomentosa* Steud. Paulowniaceae (fruits)	22.9 ± 1.6 µM ^a,f^	PHY 0.15 ± 0.03 µM ^a,f^	6.4 ± 0.9 µM ^a,m^	PHY 3.7 ± 0.6 µM ^a,m^	MCEF	
6-Geranyl-4′,5,7-trihydroxy-3′,5′-dimethoxyflavanone	*Paulownia* *tomentosa* Steud. Paulowniaceae (fruits)	316.3 ± 12.5 µM ^a,f^	PHY 0.15 ± 0.03 µM ^a,f^	80.00 ± 2.6 µM ^a,m^	PHY 3.7 ± 0.6 µM ^a,m^	MCEF	
Mimulone	*Paulownia* *tomentosa* Steud. Paulowniaceae (fruits)	91.5 ± 5.3 µM ^a,f^	PHY 0.15 ± 0.03 µM ^a,f^	20.6 ± 1.1 µM ^a,m^	PHY 3.7 ± 0.6 µM ^a,m^	MCEF	
Dihydrowogonin	*Prunus padus* var. *seoulensis* Nakai Rosaceae (leaves)	21.53 ± 0.32 µM ^a,e^	TAC 0.22 ± 0.001 µM ^a,e^	nd	nd	MCE	[48,59]
Dihydrowogonin 7-O-glucoside	*Prunus padus* var. *seoulensis* Nakai Rosaceae (leaves)	15.49 ± 0.11 µM ^a,e^	TAC 0.22 ± 0.001 µM ^a,e^	nd	nd	MCE	
Genkwanin	*Prunus padus* var. *seoulensis* Nakai Rosaceae (leaves)	17.03 ± 0.77 µM ^a,e^	TAC 0.22 ± 0.001 µM ^a,e^	nd	nd	MCE	
Rhamnocitrin	*Prunus padus* var. *seoulensis* Nakai Rosaceae (leaves)	18.26 ± 0.075 µM ^a,e^	TAC 0.22 ± 0.001 µM ^a,e^	nd	nd	MCE	
3,5,7-Trihydroxy-8-methoxyflavanone	*Prunus padus* var. *seoulensis* Nakai Rosaceae (leaves)	17.92 ± 0.63 µM ^a,e^	TAC 0.22 ± 0.001 µM ^a,e^	nd	nd	MCE	
Amentoflavone	*Selaginella doederleinii* Hieron Selaginellaceae (whole plant)	0.73 ± 0.009 µM ^a,e^	TAC 1.26 ± 0.017 µM ^a,e^	nd	nd	MCE	[48,113]
Bilobetin	*Selaginella doederleinii* Hieron Selaginellaceae (whole plant)	5.76 ± 0.021 µM ^a,e^	TAC 1.26 ± 0.017 µM ^a,e^	nd	nd	MCE	
Isoginkgetin	*Selaginella doederleinii* Hieron Selaginellaceae (whole plant)	4.11 ± 0.019 µM ^a,e^	TAC 1.26 ± 0.017 µM ^a,e^	nd	nd	MCE	
Robustaflavone	*Selaginella doederleinii* Hieron Selaginellaceae (whole plant)	6.16 ± 0.032 µM ^a,e^	TAC 1.26 ± 0.017 µM ^a,e^	nd	nd	MCE	
Kaempferol	*Spiranthes sinensis* Ames Orchidaceae (whole plant)	12.64 ± 0.31 ^a,k^	GAL 0.19 ± 0.02 µg/mL ^a,k^	nd	nd	MCE	[48,114]
Quercetin	*Spiranthes sinensis* Ames Orchidaceae (whole plant)	8.63 ± 0.37 ^a,k^	GAL 0.19 ± 0.02 µg/mL ^a,k^	nd	nd	MCE	
LANOSTANE TRITERPENES							
Methyl lucidenate E2	*Ganoderma lucidum* Karst. Ganodermataceae (fruiting bodies)	17.14 ± 2.88 µM ^a,k^	BERCl 0.04 ± 0.01 µM ^a,k^	>200 µM ^a,n^	BERCl 18.97 ± 0.41 µM ^a,n^	MCE	[48,115]
n-Butyl lucidenate A	*Ganoderma lucidum* Karst. Ganodermataceae (fruiting bodies)	12.26 ± 0.68 µM ^a,k^	BERCl 0.04 ± 0.01 µM ^a,k^	>200 µM ^a,n^	BERCl 18.97 ± 0.41 µM ^a,n^	MCE	
Ganoderic acid E	*Ganoderma lucidum* Karst. Ganodermataceae (fruiting bodies)	18.35 ± 2.95 µM ^a,k^	BERCl 0.04 ± 0.01 µM ^a,k^	>200 µM ^a,n^	BERCl 18.97 ± 0.41 µM ^a,n^	MCE	
*N*-Butyl ganoderate H	*Ganoderma lucidum* Karst. Ganodermataceae (fruiting bodies)	9.40 ± 0.88 µM ^a,k^	BERCl 0.04 ± 0.01 µM ^a,k^	>200 µM ^a,n^	BERCl 18.97 ± 0.41 µM ^a,n^	MCE	
Lucidadiol	*Ganoderma lucidum* Karst. Ganodermataceae (fruiting bodies)	31.03 ± 1.69 µM ^a,k^	BERCl 0.04 ± 0.01 µM ^a,k^	156.27 ± 6.12 µM ^a,n^	BERCl 18.97 ± 0.41 µM ^a,n^	MCE	
Lucidenic acid N	*Ganoderma lucidum* Karst. Ganodermataceae (fruiting bodies)	25.91 ± 0.89 µM ^a,k^	BERCl 0.04 ± 0.01 µM ^a,k^	188.36 ± 3.05 µM ^a,n^	BERCl 18.97 ± 0.41 µM ^a,n^	MCE	
Lucidumol B	*Ganoderma lucidum* Karst. Ganodermataceae (fruiting bodies)	16.27 ± 0.51 µM ^a,k^	BERCl 0.04 ± 0.01 µM ^a,k^	>200 µM ^a,n^	BERCl 18.97 ± 0.41 µM ^a,n^	MCE	
n-Butyl lucidenate N	*Ganoderma lucidum* Karst. Ganodermataceae (fruiting bodies)	11.58 ± 0.36 µM ^a,k^	BERCl 0.04 ± 0.01 µM ^a,k^	>200 µM ^a,n^	BERCl 18.97 ± 0.41 µM ^a,n^	MCE	
LIGNANS							
Macelignan	*Myristica fragrans* Houtt. Myristicaceae (seeds)	4.16 ± 0.070 µM ^a,e^	BER 1.01 ± 0.01 µM ^a,e^ TAC 0.22 ± 0.004 µM ^a,e^	9.69 ± 0.98 µM ^a,m^	TAC 0.014 ± 0.0043 µM ^a,m^	MCE	[48,59,60]
(+)-(7*R*,8*S*)-Erythro-4,7,9′-trihydroxy-8-O-4′-neolignan-9-O-*β*-*D*-glucopyranoside	*Camelia sinensis* var. *sinensis* Theaceae (leaves and buds)	0.75 ± 0.04 µM ^a,e^	HUP 0.29 ± 0.05 µM ^a,e^	nd	nd	MCE	[48,116,117]
(7*S*,8*S*)-Threo-4,9,9′-trihydroxy-8-O-4′-neolignan-7-O-*β*-*D*-glucopyranoside	*Camelia sinensis* var. *sinensis* Theaceae (leaves and buds)	0.19 ± 0.02 µM ^a,e^	HUP 0.29 ± 0.05 µM ^a,e^	nd	nd	MCE	
STILBENOID							
Isoarundinin II	*Bletilla striata* Reichb. f. Orchidaceae (tuber)	25 μg mL^−1^—0.9 ± 0.8% ^b,e^	GAL 25 μg mL^−1^—94.8 ± 0.9% ^b,e^	25 μg mL^−1^—39.3 ± 2.3% ^b,m^	GAL 25 μg mL^−1^—64.2 ± 0.6% ^b,m^ 46.3 ± 5.8 µM ^a,m^ TAC 0.0101 ± 0.0005 µM ^a,m^	MCE	[37,48]
PHENANTHRENES							
1-[(4-Hydroxyphenyl)methyl]-4-methoxy-2,7-phenanthrenediol	*Bletilla striata* Reichb. f. Orchidaceae (tuber)	25 μg mL^−1^—19.1 ± 3.8% ^b,e^	GAL 25 μg mL^−1^—94.8 ± 0.9% ^b,e^	25 μg mL^−1^—96.6 ± 1.2% ^b,m^ 2.1 ± 0.3 µM ^a,m^	GAL 25 μg mL^−1^—64.2 ± 0.6% ^b,m^ 46.3 ± 5.8 µM ^a,m^ TAC 0.0101 ± 0.0005 µM ^a,m^	MCE	[37,48]
1,8-bis(4-Hydroxybenzyl)-4-methoxyphenanthrene-2,7-diol	*Bletilla striata* Reichb. f. Orchidaceae (tuber)	25 μg mL^−1^—16.1 ± 5.0% ^b,e^	GAL 25 μg mL^−1^—94.8 ± 0.9% ^b,e^	25 μg mL^−1^—95.4 ± 0.3% ^b,m^ 2.3 ± 0.4 µM ^a,m^	GAL 25 μg mL^−1^—64.2 ± 0.6% ^b,m^ 46.3 ± 5.8 µM ^a,m^ TAC 0.0101 ± 0.0005 µM ^a,m^	MCE	
2,7-Dihydroxy-1,3-bi(p-hydroxybenzyl)-4-methoxy-9,10-dihydrophenanthrene	*Bletilla striata* Reichb. f. Orchidaceae (tuber)	25 μg mL^−1^—20.1 ± 3.5% ^b,e^	GAL 25 μg mL^−1^—94.8 ± 0.9% ^b,e^	25 μg mL^−1^—53.1 ± 1.2% ^b,m^ 44.6 ± 4.1 µM ^a,m^	GAL 25 μg mL^−1^—64.2 ± 0.6% ^b,m^ 46.3 ± 5.8 µM ^a,m^ TAC 0.0101 ± 0.0005 µM ^a,m^	MCE	
1-(*p*-Hydroxybenzyl)-4, 7-dimethoxyphenanthrene-2,8-diol	*Bletilla striata* Reichb. f. Orchidaceae (tuber)	25 μg mL^−1^—20.4 ± 4.5% ^b,e^	GAL 25 μg mL^−1^—94.8 ± 0.9% ^b,e^	25 μg mL^−1^—85.2 ± 2.9% ^b,m^ 6.4 ± 0.2 µM ^a,m^	GAL 25 μg mL^−1^—64.2 ± 0.6% ^b,m^ 46.3 ± 5.8 µM ^a,m^ TAC 0.0101 ± 0.0005 µM ^a,m^	MCE	
3-(4-Hydroxybenzyl)-4-methoxy-9,10-dihydrophenanthrene-2,7-diol	*Bletilla striata* Reichb. f. Orchidaceae (tuber)	25 μg mL^−1^—9.6 ± 2.6% ^b,e^	GAL 25 μg mL^−1^—94.8 ± 0.9% ^b,e^	25 μg mL^−1^—65.7 ± 0.7% ^b,m^ 34.0 ± 1.4 µM ^a,m^	GAL 25 μg mL^−1^—64.2 ± 0.6% ^b,m^ 46.3 ± 5.8 µM ^a,m^ TAC 0.0101 ± 0.0005 µM ^a,m^	MCE	
9-(4′-Hydroxy-3′- methoxyphenyl)-10-(hydroxymethyl)-11-methoxy-5,6,9, 10-tetrahydrophenanthro [2,3-b] furan-3-ol	*Bletilla striata* Reichb. f. Orchidaceae (tuber)	25 μg mL^−1^—3.3 ± 1.8% ^b,e^	GAL 25 μg mL^−1^—94.8 ± 0.9% ^b,e^	25 μg mL^−1^—61.2 ± 1.3% ^b,m^ 35.8 ± 9.2 µM ^a,m^	GAL 25 μg mL^−1^—64.2 ± 0.6% ^b,m^ 46.3 ± 5.8 µM ^a,m^ TAC 0.0101 ± 0.0005 µM ^a,m^	MCE	
Bleformin A	*Bletilla striata* Reichb. f. Orchidaceae (tuber)	25 μg mL^−1^—18.5 ± 1.7% ^b,e^	GAL 25 μg mL^−1^—94.8 ± 0.9% ^b,e^	25 μg mL^−1^—70.0 ± 1.0% ^b,m^ 5.2 ± 0.4 µM ^a,m^	GAL 25 μg mL^−1^—64.2 ± 0.6% ^b,m^ 46.3 ± 5.8 µM ^a,m^ TAC 0.0101 ± 0.0005 µM ^a,m^	MCE	
Bleformin B	*Bletilla striata* Reichb. f. Orchidaceae (tuber)	25 μg mL^−1^—9.9 ± 4.7% ^b,e^	GAL 25 μg mL^−1^—94.8 ± 0.9% ^b,e^	25 μg mL^−1^—75.7 ± 1.1% ^b,m^ 16.7 ± 2.4 µM ^a,m^	GAL 25 μg mL^−1^—64.2 ± 0.6% ^b,m^ 46.3 ± 5.8 µM ^a,m^ TAC 0.0101 ± 0.0005 µM ^a,m^	MCE	
Blestrin D	*Bletilla striata* Reichb. f. Orchidaceae (tuber)	25 μg mL^−1^—6.8 ± 1.6% ^b,e^	GAL 25 μg mL^−1^—94.8 ± 0.9% ^b,e^	25 μg mL^−1^—69.0 ± 2.5% ^b,m^ 8.1 ± 0.5 µM ^a,m^	GAL 25 μg mL^−1^—64.2 ± 0.6% ^b,m^ 46.3 ± 5.8 µM ^a,m^ TAC 0.0101 ± 0.0005 µM ^a,m^	MCE	
Blestrin A	*Bletilla striata* Reichb. f. Orchidaceae (tuber)	25 μg mL^−1^—8.4 ± 3.1% ^b,e^	GAL 25 μg mL^−1^—94.8 ± 0.9% ^b,e^	25 μg mL^−1^—64.0 ± 2.6% ^b,m^ 17.9 ± 4.7 µM ^a,m^	GAL 25 μg mL^−1^—64.2 ± 0.6% ^b,m^ 46.3 ± 5.8 µM ^a,m^ TAC 0.0101 ± 0.0005 µM ^a,m^	MCE	
Blestrin C	*Bletilla striata* Reichb. f. Orchidaceae (tuber)	25 μg mL^−1^—4.9 ± 3.2% ^b,e^	GAL 25 μg mL^−1^—94.8 ± 0.9% ^b,e^	25 μg mL^−1^—64.3 ± 2.4% ^b,m^ 12.1 ± 3.4 µM ^a,m^	GAL 25 μg mL^−1^—64.2 ± 0.6% ^b,m^ 46.3 ± 5.8 µM ^a,m^ TAC 0.0101 ± 0.0005 µM ^a,m^	MCE	
Bletilol D	*Bletilla striata* Reichb. f. Orchidaceae (tuber)	25 μg mL^−1^—5.7 ± 2.8% ^b,e^	GAL 25 μg mL^−1^—94.8 ± 0.9% ^b,e^	25 μg mL^−1^—31.6 ± 2.8% ^b,m^	GAL 25 μg mL^−1^—64.2 ± 0.6% ^b,m^ 46.3 ± 5.8 µM ^a,m^ TAC 0.0101 ± 0.0005 µM ^a,m^	MCE	
Bletilol E	*Bletilla striata* Reichb. f. Orchidaceae (tuber)	25 μg mL^−1^—5.1 ± 4.0% ^b,e^	GAL 25 μg mL^−1^—94.8 ± 0.9% ^b,e^	25 μg mL^−1^—8.0 ± 2.4% ^b,m^	GAL 25 μg mL^−1^—64.2 ± 0.6% ^b,m^ 46.3 ± 5.8 µM ^a,m^ TAC 0.0101 ± 0.0005 µM ^a,m^	MCE	
Favanthrin	*Bletilla striata* Reichb. f. Orchidaceae (tuber)	25 μg mL^−1^—13.3 ± 2.9% ^b,e^	GAL 25 μg mL^−1^—94.8 ± 0.9% ^b,e^	25 μg mL^−1^—56.7 ± 2.0% ^b,m^ 42.2 ± 5.1 µM ^a,m^	GAL 25 μg mL^−1^—64.2 ± 0.6% ^b,m^ 46.3 ± 5.8 µM ^a,m^ TAC 0.0101 ± 0.0005 µM ^a,m^	MCE	
Pholidotol	*Bletilla striata* Reichb. f. Orchidaceae (tuber)	25 μg mL^−1^ –5.2 ± 3.2% ^b,e^	GAL 25 μg mL^−1^—94.8 ± 0.9% ^b,e^	25 μg mL^−1^—29.1 ± 1.3% ^b,m^	GAL 25 μg mL^−1^—64.2 ± 0.6% ^b,m^ 46.3 ± 5.8 µM ^a,m^ TAC 0.0101 ± 0.0005 µM ^a,m^	MCE	
Shancidin	*Bletilla striata* Reichb. f. Orchidaceae (tuber)	25 μg mL^−1^—15.2 ± 3.6% ^b,e^	GAL 25 μg mL^−1^—94.8 ± 0.9% ^b,e^	25 μg mL^−1^—72.8 ± 3.4% ^b,m^ 16.7 ± 2.0 µM ^a,m^	GAL 25 μg mL^−1^—64.2 ± 0.6% ^b,m^ 46.3 ± 5.8 µM ^a,m^ TAC 0.0101 ± 0.0005 µM ^a,m^	MCE	
Shanciol F	*Bletilla striata* Reichb. f. Orchidaceae (tuber)	25 μg mL^−1^—5.5 ± 1.8% ^b,e^	GAL 25 μg mL^−1^—94.8 ± 0.9% ^b,e^	25 μg mL^−1^—21.8 ± 3.1% ^b,m^	GAL 25 μg mL^−1^—64.2 ± 0.6% ^b,m^ 46.3 ± 5.8 µM ^a,m^ TAC 0.0101 ± 0.0005 µM ^a,m^	MCE	
Cremaphenanthrene F	*Cremastra appendiculata* Makino Orchidaceae (tubers)	>200 µM ^a,e^	GAL 0.39 ± 0.04 µM ^a,e^	14.62 ± 2.15 µM ^a,m^	GAL 1.12 ± 0.67 µM ^a,m^	MCE	[44,48]
Cremaphenanthrene G	*Cremastra appendiculata* Makino Orchidaceae (tubers)	>200 µM ^a,e^	GAL 0.39 ± 0.04 µM ^a,e^	79.56 ± 0.78 µM ^a,m^	GAL 1.12 ± 0.67 µM ^a,m^	MCE	
PHENYLPROPANOIDS							
Lapathoside A	*Fallopia dentatoalata* Holub Polygonaceae (aerial part)	30.6 ± 4.7 µM ^a,e^	TAC 0.1267 ± 0.0011 µM ^a,e^	2.7 ± 1.7 µM ^a,m^	TAC 0.0055 ± 0.0017 µM ^a,m^	MCE	[48,118,119]
Lapathoside B	*Fallopia dentatoalata* Holub Polygonaceae (aerial part)	>100 µM ^a,e^	TAC 0.1267 ± 0.0011 µM ^a,e^	10.9 ± 4.9 µM ^a,m^	TAC 0.0055 ± 0.0017 µM ^a,m^	MCE	
Smilaside G	*Fallopia dentatoalata* Holub Polygonaceae (aerial part)	>100 µM ^a,e^	TAC 0.1267 ± 0.0011 µM ^a,e^	17.1 ± 3.4 µM ^a,m^	TAC 0.0055 ± 0.0017 µM ^a,m^	MCE	
Smilaside J	*Fallopia dentatoalata* Holub Polygonaceae (aerial part)	56.0 ± 2.4 µM ^a,e^	TAC 0.1267 ± 0.0011 µM ^a,e^	10.1 ± 4.6 µM ^a,m^	TAC 0.0055 ± 0.0017 µM ^a,m^	MCE	
Vanicoside B	*Fallopia dentatoalata* Holub Polygonaceae (aerial part)	32.3 ± 4.7µM ^a,e^	TAC 0.1267 ± 0.0011 µM ^a,e^	7.5 ± 4.1 µM ^a,m^	TAC 0.0055 ± 0.0017 µM ^a,m^	MCE	
PHLOROTANNINS							
974-B	*Eisenia bicyclis* (Kjellman) Stechell Laminariaceae (leafy thalli)	1.95 ± 0.01 μM ^a,e^	BER 0.22 ± 0.03 µM ^a,e^	3.26 ± 0.08 µM ^a,m^	BER 11.74 ± 0.85 µM ^a,m^	CE	[48,120]
PHTHALATES							
bis (7-Acetoxy-2-ethyl- 5-methylheptyl) phthalate	*Lonicera quinquelocularis* Hard. Caprifoliaceae (whole plant)	1.65 ± 0.03 µM ^a,k^	GAL 1.79 ± 0.061 µM ^a,k^	5.98 ± 0.079 µM ^a,m^	GAL 7.98 ± 0.01 µM ^a,m^	MCE	[48,51,121]
Neopentyl-4-hydroxy-3,5-bis (3-methyl-2-butenyl) benzoate	*Lonicera quinquelocularis* Hard. Caprifoliaceae (whole plant)	3.43 ± 0.02 µM ^a,k^	GAL 1.79 ± 0.061 µM ^a,k^	9.84 ± 0.037 µM ^a,m^	GAL 7.98 ± 0.01 µM ^a,m^	MCE	
PHENOLIC ACIDS							
4-Hydroxybenzoic acid methyl ester	*Spiranthes sinensis* Ames Orchidaceae (whole plant)	42.89 ± 1.21 ^a,k^	GAL 0.19 ± 0.02 µg/mL ^a,k^	nd	nd	MCE	[48,114]
Ethyl ferulate	*Spiranthes sinensis* Ames Orchidaceae (whole plant)	19.97 ± 1.05 ^a,k^	GAL 0.19 ± 0.02 µg/mL ^a,k^	nd	nd	MCE	
3-(4-Tolyloxy)-propanoic acid	*Spiranthes sinensis* Ames Orchidaceae (whole plant)	15.31 ± 0.64 ^a,k^	GAL 0.19 ± 0.02 µg/mL ^a,k^	nd	nd	MCE	
POLYKETIDES							
Aspilactonol G	*Phaeospaeria* sp. LF5 (strain from Huperzia serrata)	>100 µM ^a,k^	RIV 1.82 ± 0.13 µM ^a,k^ HUP 0.045 ± 0.01 µM ^a,k^	nd	nd	MCE	[48,122,123]
Aspilactonol H	*Phaeospaeria* sp. LF5 (strain from Huperzia serrata)	>100 µM ^a,k^	RIV 1.82 ± 0.13 µM ^a,k^ HUP 0.045 ± 0.01 µM ^a,k^	nd	nd	MCE	
Aspilactonol I	*Phaeospaeria* sp. LF5 (strain from Huperzia serrata)	6.26 ± 0.15 µM ^a,k^	RIV 1.82 ± 0.13 µM ^a,k^ HUP 0.045 ± 0.01 µM ^a,k^	nd	nd	MCE	
de-O-Methyldiaporthin	*Phaeospaeria* sp. LF5 (strain from Huperzia serrata)	21.18 ± 1.53 µM ^a,k^	RIV 1.82 ± 0.13 µM ^a,k^ HUP 0.045 ± 0.01 µM ^a,k^	nd	nd	MCE	
6,8-Dihydroxy-3-(1*0R*, 2*0R*-dihydroxypropyl)-isocoumarin	*Phaeospaeria* sp. LF5 (strain from Huperzia serrata)	>100 µM ^a,k^	RIV 1.82 ± 0.13 µM ^a,k^ HUP 0.045 ± 0.01 µM ^a,k^	nd	nd	MCE	
*E*-Δ^2^-Anhydromevalonic acid	*Phaeospaeria* sp. LF5 (strain from Huperzia serrata)	>100 µM ^a,k^	RIV 1.82 ± 0.13 µM ^a,k^ HUP 0.045 ± 0.01 µM ^a,k^	nd	nd	MCE	
2-(1-Hydroxyethyl)-6- methylisonicotinic acid	*Phaeospaeria* sp. LF5 (strain from Huperzia serrata)	>100 µM ^a,k^	RIV 1.82 ± 0.13 µM ^a,k^ HUP 0.045 ± 0.01 µM ^a,k^	nd	nd	MCE	
6-Hydroxy-8-methoxy-3- methylisocoumarin	*Phaeospaeria* sp. LF5 (strain from Huperzia serrata)	>100 µM ^a,k^	RIV 1.82 ± 0.13 µM ^a,k^ HUP 0.045 ± 0.01 µM ^a,k^	nd	nd	MCE	
3-(Hydroxymethyl)-5-methylfuran-2(5H)-one	*Phaeospaeria* sp. LF5 (strain from Huperzia serrata)	>100 µM ^a,k^	RIV 1.82 ± 0.13 µM ^a,k^ HUP 0.045 ± 0.01 µM ^a,k^	nd	nd	MCE	
4-Methyl-5,6-dihydropyren-2-one	*Phaeospaeria* sp. LF5 (strain from Huperzia serrata)	>100 µM ^a,k^	RIV 1.82 ± 0.13 µM ^a,k^ HUP 0.045 ± 0.01 µM ^a,k^	nd	nd	MCE	
(*R*)-6-Hydroxymellein	*Phaeospaeria* sp. LF5 (strain from Huperzia serrata)	>100 µM ^a,k^	RIV 1.82 ± 0.13 µM ^a,k^ HUP 0.045 ± 0.01 µM ^a,k^	nd	nd	MCE	
Asterric acid	*Talaromyces aurantiacus* FL 15 (strain from leave *Huperzia serrata*)	66.7 ± 1.7 µM ^a,e^	RIV 1.82 ± 0.13 µM ^a,e^ HUP 0.045 ± 0.01 µM ^a,e^	>100 µM ^a,m^	ns	MCE	[48,91,92]
Ethyl asterrate	*Talaromyces aurantiacus* FL 15 (strain from leave *Huperzia serrata*)	20.1 ± 0.9 µM ^a,e^	RIV 1.82 ± 0.13 µM ^a,e^ HUP 0.045 ± 0.01 µM ^a,e^	>100 µM ^a,m^	ns	MCE	
Methyl asterrate	*Talaromyces aurantiacus* FL 15 (strain from leave *Huperzia serrata*)	23.3 ± 1.2 µM ^a,e^	RIV 1.82 ± 0.13 µM ^a,e^ HUP 0.045 ± 0.01 µM ^a,e^	>100 µM ^a,m^	ns	MCE	
Sulochrin	*Talaromyces aurantiacus* FL 15 (strain from leave *Huperzia serrata*)	>100 µM ^a,e^	RIV 1.82 ± 0.13 µM ^a,e^ HUP 0.045 ± 0.01 µM ^a,e^	>100 µM ^a,m^	ns	MCE	
POLYPHENOLS							
Broussonin A	*Anemarrhena asphodeloides* Bunge Asparagaceae (roots)	15.88 ± 1.02 µM ^a,e^	BER 1.01 ± 0.01 µM ^a,e^ TAC 0.22 ± 0.004 µM ^a,e^	7.50 ± 0.07 µM ^a,m^	TAC 0.014 ± 0.0043 µM ^a,m^	MCE	[48,59,60]
Mangiferin	*Anemarrhena asphodeloides* Bunge Asparagaceae (whole plant)	62.8 µM ^a,g^	TAC nd ^a,g^	nd	nd	MCE	[48,124]
Caffeoylated catechin	*Camellia sinensis* var. *assamica* Theaceae (leaves)	2.49 ± 0.43 µM ^a,e^	HUP 0.088 ± 0.004 µM ^a,e^	nd	d	MCE	[48,116]
Epigallocatechin 3-O-*p*-coumaroate	*Camellia sinensis* var. *assamica* Theaceae (leaves)	11.41 ± 2.00 µM ^a,e^	HUP 0.088 ± 0.004 µM ^a,e^	nd	nd	MCE	
Epigallocatechin-3-O-ferulate	*Camellia sinensis* var. *assamica* Theaceae (leaves)	62.26 ± 10.18 µM ^a,e^	HUP 0.088 ± 0.004 µM ^a,e^	nd	nd	MCE	
Creoside IV	*Codonopsis pilosula* Nannf Campanulaceae (roots)	7.30 ± 0.49 µM ^a,e^	BER 1.01 ± 0.01 µM ^a,e^ TAC 0.22 ± 0.004 µM ^a,e^	>40 ^a,m^	TAC 0.014 ± 0.0043 µM ^a,m^	MCE	[48,59,60]
Heyneanol A	*Vitis amurensis* Rupr. Vitaceae (roots)	1.66 ± 0.09 µM ^a,f^	GAL 0.93 ± 0.07 µM ^a,f^	1.75 ± 0.09 µM ^a,l^	GAL 9.24 ± 1.32 µM ^a,l^	MCE	[48,125]
Vitisin A	*Vitis amurensis* Rupr. Vitaceae (roots)	1.04 ± 0.05 µM ^a,f^	GAL 0.93 ± 0.07 µM ^a,f^	4.41 ± 0.39 µM ^a,l^	GAL 9.24 ± 1.32 µM ^a,l^	MCE	
SESQUITERPENE LACTONES							
Britannin	*Inula aucheriana* DC. Asteraceae (aerial parts)	300 μg mL^−1^—25.2% ^b,k^	DON	nd	nd	MCE	[48,126]
Gaillardin	*Inula oculus-christi* L. Asteraceae (aerial parts)	300 μg mL^−1^—67% ^b,k^	DON	nd	nd	MCE	
Pulchellin C	*Inula oculus-christi* L*.* Asteraceae (aerial parts)	300 μg mL^−1^—10.9% ^b,k^	DON	nd	nd	MCE	
Amberin	*Amberboa ramosa* Jafri. Asteraceae (whole plant)	17.5 ± 0.01 μM ^a,e^	GAL 0.5 ± 0.01 μM ^a,e^ PHY 0.04 ± 0.0001 μM ^a,e^	2.7 ± 0.02 μM ^a,m^	GAL 8.2 ± 0.02 μM ^a,m^ PHY 0.82 ± 0.001 μM ^a,m^	MCE	[48,127]
Amberbin A	*Amberboa ramosa* Jafri. Asteraceae (whole plant)	8.6 ± 0.15 μM ^a,e^	GAL 0.5 ± 0.01 μM ^a,e^ PHY 0.04 ± 0.0001 μM ^a,e^	4.8 ± 0.15 μM ^a,m^	GAL 8.2 ± 0.02 μM ^a,m^ PHY 0.82 ± 0.001 μM ^a,m^	MCE	
Amberbin B	*Amberboa ramosa* Jafri. Asteraceae (whole plant))	0.91 ± 0.015 μM ^a,e^	GAL 0.5 ± 0.01 μM ^a,e^ PHY 0.04 ± 0.0001 μM ^a,e^	2.5 ± 0.15 μM ^a,m^	GAL 8.2 ± 0.02 μM ^a,m^ PHY 0.82 ± 0.001 μM ^a,m^	MCE	
Amberbin C	*Amberboa ramosa* Jafri. Asteraceae (whole plant)	1.1 ± 0.08 μM ^a,e^	GAL 0.5 ± 0.01 μM ^a,e^ PHY 0.04 ± 0.0001 μM ^a,e^	17.9 ± 0.05 μM ^a,m^	GAL 8.2 ± 0.02 μM ^a,m^ PHY 0.82 ± 0.001 μM ^a,m^	MCE	
Zerumbone	*Zingiber zerumbet* L. Zingiberaceae (whole plant)	1 mg mL^−1 c,k^	TAC 10 mM ^c,k^	nd	nd	BTLC by Rhee et al. (2001)	[16,128]
Silphiperfolene acetate	*Leontopodium alpinum* Cass. Asteraceae (sub-aerial parts)	200 μM—40.64 ± 7.09% ^b,k^	GAL 3.2 µM ^a,k^ GAL 100 μM—89.30 ± 2.29% ^b,k^	nd	nd	MCE	[93,95,129]
STEROIDS							
Leucisterol	*Leucas urticifolia* Vahl. Lamiaceae (whole plant)	83.6 ± 0.59 µM ^a,k^	PHY 0.04 µM ^a,k^	3.2 ± 0.85 µM ^a,n^	PHY 0.93 ± 0.3 µM ^a,n^	CE	[48,130]
STEROLS							
Haloxylon A	*Haloxylon recurvum* Bunge ex Boiss Chenopodiaceae (whole plant)	8.3 ± 0.02 µM ^a,e^	GAL 0.5 ± 0.001 µM ^a,e^	4.7 ± 0.01 µM ^a,m^	GAL 8.5 ± 0.00 µM ^a,m^	MCE	[48,131]
Haloxylon B	*Haloxylon recurvum* Bunge ex Boiss Chenopodiaceae (whole plant)	0.89 ± 0.002 µM ^a,e^	GAL 0.5 ± 0.001 µM ^a,e^	2.3 ± 0.001 µM ^a,m^	GAL 8.5 ± 0.00 µM ^a,m^	MCE	
TRIFLAVANONES							
Garcineflavanone A	*Garcinia atroviridis* Griff. ex T. Anders. Clusiaceae (stem bark)	100 μg mL^−1^—80.15 ± 6.65% ^b,e^ 28.52 ± 5.23 μg mL^−1 a,e^	PHY 0.05 ± 0.01 μg mL^−1 a,e^	ns	PHY 0.14 ± 0.015 μg mL^−1 a,m^	MCE	[48,108,109]
TRITERPENOIDS							
Arbora- 1,9(11)-dien-3-one	*Buxus hyrcana* Pojark. Buxaceae (leaves)	47.9 ± 1.2 µM ^a,k^	GAL 0.53 ± 0.5 µM ^a,k^ HUP 1.7 ± 0.3 µM ^a,k^	220.1 ± 1.0 µM ^a,n^	GAL 8.7 ± 1.0 µM ^a,n^ HUP >1000 ± 3.0 µM ^a,n^	MCE	[48,56,57,58]
Asiatic acid	*Centella asiatica* Urb Apiaceae (whole plant)	15.05 ± 0.05 µM ^a,e^	PHY 0.05 ± 0.12 µM ^a,e^	nd	nd	MCE	[48,132,133]
Asiaticoside	*Centella asiatica* Urb Apiaceae (whole plant)	59.13 ± 0.18 µM ^a,e^	PHY 0.05 ± 0.12 µM ^a,e^	nd	nd	MCE	
Madecassic acid	*Centella asiatica* Urb Apiaceae (whole plant)	17.83 ± 0.06 µM ^a,e^	PHY 0.05 ± 0.12 µM ^a,e^	nd	nd	MCE	
Madecassoside	*Centella asiatica* Urb Apiaceae (whole plant)	37.14 ± 0.04 µM ^a,e^	PHY 0.05 ± 0.12 µM ^a,e^	nd	nd	MCE	
Betulin	*Garcinia hombroniana* Pierre Clusiaceae (bark)	28.5 ± 0.78 µM ^a,e^	PHY 0.04 ± 0.004 µM ^a,e^	nd	PHY 0.09 ± 0.003 µM ^a,m^	MCE	[48,81]
Betulinic acid	*Garcinia hombroniana* Pierre Clusiaceae (bark)	24.2 ± 0.99 µM ^a,e^	PHY 0.04 ± 0.004 µM ^a,e^	19.1 ± 1.33 µM ^a,m^	PHY 0.09 ± 0.003 µM ^a,m^	MCE	
2*β*-Hydroxy-3*α*-*O*-caffeoyltaraxar-14-en-28- oic acid	*Garcinia hombroniana* Pierre Clusiaceae (bark)	13.5 ± 0.95 µM ^a,e^	PHY 0.04 ± 0.004 µM ^a,e^	10.6 ± 0.54 µM ^a,m^	PHY 0.09 ± 0.003 µM ^a,m^	MCE	
Taraxerol	*Garcinia hombroniana* Pierre Clusiaceae (bark)	nd	PHY 0.04 ± 0.004 µM ^a,e^	17.8 ± 1.73 µM ^a,m^	PHY 0.09 ± 0.003 µM ^a,m^	MCE	
21β-Hydroxyserrat- 14-en-3,16-dione	*Lycopodiella cernua* L. Lycopodiaceae*)* (whole plants)	10.67 ± 0.66 µM ^a,k^	BER 0.10 ± 0.01 µM ^a,k^	>30 µM ^a,n^	BER 1.09 ± 0.17 µM ^a,n^	MCE	[48,106]
3β,21α-Diacetoxyserratan- 14β-ol	*Lycopodiella cernua* L. Lycopodiaceae (whole plants)	0.91 ± 0.01 µM ^a,k^	BER 0.10 ± 0.01 µM ^a,k^	>30 µM ^a,n^	BER 1.09 ± 0.17 µM ^a,n^	MCE	
3β,21β,29-Trihydroxyserrat- 14-en-3β-yl p-dihydrocoumarate	*Lycopodiella cernua* L. Lycopodiaceae (whole plants)	1.69 ± 0.10 µM ^a,k^	BER 0.10 ± 0.01 µM ^a,k^	0.42 ± 0.01 µM ^a,n^	BER 1.09 ± 0.17 µM ^a,n^	MCE	
SESQUITERPENES							
1α-Acetoxy-6β,9β-difuroyloxy-4β-hydroxydihydro-β-agarofuran	*Maytenus disticha* Urb. Celastraceae (seeds)	738.0 ± 0.007 µM ^a,e^	GAL 10.0 ± 0.015 µM ^a,e^ CAR 45.0 ± 0.031 µM ^a,e^	ns ^a,m^	ns ^a,m^	MCE	[48,134]
6β-Acetoxy-9β-benzyloxy-1α,8α-dihydroxydihydro-β-agarofuran	*Maytenus disticha* Urb. Celastraceae (seeds)	500.0 ± 0.03 µM ^a,e^	GAL 10.0 ± 0.015 µM ^a,e^ CAR 45.0 ± 0.031 µM ^a,e^	ns ^a,m^	ns ^a,m^	MCE	
6β,8α-Diacetoxy-9β-furoyloxy-1α-hydroxydihydro-β-agarofuran	*Maytenus disticha* Urb. Celastraceae (seeds)	740.0 ± 0.045 µM ^a,e^	GAL 10.0 ± 0.015 µM ^a,e^ CAR 45.0 ± 0.031 µM ^a,e^	ns ^a,m^	ns ^a,m^	MCE	
1α,6β,14-Triacetoxy-9β-benzyloxydihydro-β-agarofuran	*Maytenus magellanica* Hook.f. Celastraceae (seeds)	695.0 ± 0.001 µM ^a,e^	GAL 10.0 ± 0.015 µM ^a,e^ CAR 45.0 ± 0.031 µM ^a,e^	ns ^a,m^	ns ^a,m^	MCE	
2α,3β,6β-Triacetoxy-1α,9β-dibenzyloxy-4β-hydroxydihydro-β-agarofuran	*Maytenus magellanica* Hook.f. Celastraceae (seeds)	30.0 ± 0.06 µM ^a,e^	GAL 10.0 ± 0.015 µM ^a,e^ CAR 45.0 ± 0.031 µM ^a,e^	ns ^a,m^	ns ^a,m^	MCE	
XANTHONES							
Bellidin	*Gentianella amarella* ssp. *acuta* J.M.Gillett Gentianaceae (whole plants)	10 μM—17.5 ± 5.7% ^b,e^	GAL 10 μM—96.82 ± 0.04% ^b,e^	nd	nd	MCE BTLC by Marston et al. (2002)	[42,48,101,135]
Bellidifolin	*Gentianella amarella* ssp. *acuta* J.M.Gillett Gentianaceae (whole plants)	10 μM—21.9 ± 6.2% ^b,e^	GAL 10 μM—96.82 ± 0.04% ^b,e^	nd	nd	MCE BTLC by Marston et al. (2002)	
Corymbiferin 1-O-glucoside	*Gentianella amarella* ssp. *acuta* J.M.Gillett Gentianaceae (whole plants)	10 μM—1.5 ± 1.2% ^b,e^	GAL 10 μM—96.82 ± 0.04% ^b,e^	nd	nd	MCE BTLC by Marston et al. (2002)	
Corymbiferin 3-O-β-D-glucopyranoside	*Gentianella amarella* ssp. *acuta* J.M.Gillett Gentianaceae (whole plants)	10 μM—17.6 ± 1.8% ^b,e^	GAL 10 μM—96.82 ± 0.04% ^b,e^	nd	nd	MCE BTLC by Marston et al. (2002)	
*nor*-Swertianolin	*Gentianella amarella* ssp. *acuta* J.M.Gillett Gentianaceae (whole plants)	10 μM—4.4 ± 4.4% ^b,e^	GAL 10 μM—96.82 ± 0.04% ^b,e^	nd	nd	MCE BTLC by Marston et al. (2002)	
Swertianolin	*Gentianella amarella* ssp. *acuta* J.M.Gillett Gentianaceae (whole plants)	10 μM—9.8 ± 3.9% ^b,e^	GAL 10 μM—96.82 ± 0.04% ^b,e^	nd	nd	MCE BTLC by Marston et al. (2002)	
Swertiabisxanthone-I	*Gentianella amarella* ssp. *acuta* J.M.Gillett Gentianaceae (whole plants)	10 μM—20.9 ± 3.3% ^b,e^	GAL 10 μM—96.82 ± 0.04% ^b,e^	nd	nd	MCE BTLC by Marston et al. (2002)	
Swertiabisxanthone-I 8′-O-β-D-glucopyranoside	*Gentianella amarella* ssp. *acuta* J.M.Gillett Gentianaceae (whole plants)	10 μM—12.3 ± 2.9% ^b,e^	GAL 10 μM—96.82 ± 0.04% ^b,e^	nd	nd	MCE BTLC by Marston et al. (2002)	
Triptexanthoside C	*Gentianella amarella* ssp. *acuta* J.M.Gillett Gentianaceae (whole plants)	10 μM—43.7 ± 3.3% ^b,e^ 13.8 ± 1.6 µM ^a,e^	GAL 10 μM—96.82 ± 0.04% ^b,e^ GAL 0.35 ± 0.02 µM ^a,e^	nd	nd	MCE BTLC by Marston et al. (2002)	
Veratriloside	*Gentianella amarella* ssp. *acuta* J.M.Gillett Gentianaceae (whole plants)	10 μM—28.2 ± 2.5% ^b,e^	GAL 10 μM—96.82 ± 0.04% ^b,e^	nd	nd	MCE BTLC by Marston et al. (2002)	
XANTHONOIDS							
Allanxanthone E	*Garcinia mangostana* L. Clusiaceas (seedcases)	15.0 ± 1.2 µM ^a,f^ 67.4 ± 0.3 µM ^a,e^	PHY 0.043 ± 0.002 µM ^a,f^ 0.049 ± 0.003 µM ^a,e^	11.0 ± 0.4 µM ^a,m^	PHY 0.073 ± 0.006 µM ^a,m^	MCEF	[48,112,136]
α-Mangostin	*Garcinia mangostana* L. Clusiaceas (seedcases)	8.0 ± 0.5 µM ^a,f^ 6.3 ± 0.6 µM ^a,e^	PHY 0.043 ± 0.002 µM ^a,f^ 0.049 ± 0.003 µM ^a,e^	2.9 ± 0.7 µM ^a,m^	PHY 0.073 ± 0.006 µM ^a,m^	MCEF	
8-Deoxygartanin	*Garcinia mangostana* L. Clusiaceas (seedcases)	6.2 ± 0.3 µM ^a^,^f^ 11.0 ± 0.6 µM ^e^	PHY 0.043 ± 0.002 µM ^a,f^ 0.049 ± 0.003 µM ^a,e^	9.2 ± 0.5 µM ^a,m^	PHY 0.073 ± 0.006 µM ^a,m^	MCEF	
γ-Mangostin	*Garcinia mangostana* L. Clusiaceas (seedcases)	5.4 ± 0.3 µM ^a,f^ 2.5 ± 3.3 µM ^a,e^	PHY 0.043 ± 0.002 µM ^a,f^ 0.049 ± 0.003 µM ^a,e^	0.7 ± 0.03 µM ^a,m^	PHY 0.073 ± 0.006 µM ^a,m^	MCEF	
Gudraxanthone	*Garcinia mangostana* L. Clusiaceas (seedcases)	11.7 ± 0.7 µM ^a,f^ 18.9 ± 1.7 µM ^a,e^	PHY 0.043 ± 0.002 µM ^a,f^ 0.049 ± 0.003 µM ^a,e^	9.0 ± 1.2 µM ^a,m^	PHY 0.073 ± 0.006 µM ^a,m^	MCEF	
9-Hydroxy-calabaxanthone	*Garcinia mangostana* L. Clusiaceas (seedcases)	>100 µM ^a,f^ >100 µM ^a,e^	PHY 0.043 ± 0.002 µM ^a,f^ 0.049 ± 0.003 µM ^a,e^	86.3 ± 2.4 µM ^a,m^	PHY 0.073 ± 0.006 µM ^a,m^	MCEF	
Mangostanol	*Garcinia mangostana* L. Clusiaceas (seedcases)	14.6 ± 0.7 µM ^a f^ 6.3 ± 5.4 µM ^a,e^	PHY 0.043 ± 0.002 µM ^a,f^ 0.049 ± 0.003 µM ^a,e^	6.0 ± 0.2 µM ^a,m^	PHY 0.073 ± 0.006 µM ^a,m^	MCEF	
MISCELLANOUS							
3-Methylbuthyl hydrodisulfide	*Buthus martensii* Karsch Buthidae (whole body of scorpion)	40.93 ± 3.21 µM ^a,e^	GAL 1.17 ± 0.01 µM ^a,e^ DON 0.049 ± 0.004 µM ^a,e^	152.84 ± 7.22 µM ^a,m^	GAL 18.78 ± 1.81 µM ^a,m^ DON 5.536± 0.018 µM ^a,m^	MCE	[48,54,55]
2-Benzothiazolol	*Spiranthes sinensis* Ames Orchidaceae (whole plant)	37.67 ± 0.52 ^a,k^	GAL 0.19 ± 0.02 µg/mL ^a,k^	nd	nd	MCE	[48,114]

Abbreviations in Table 1: nd—not determined; ns—not shown; ^a^—inhibitory concentration for which enzyme activity is equal to half-maximal (IC_50_)/(IC_50_) ± S.E.M.; ^b^—percentage of inhibition against enzyme (xμg mL^−1^-y%, xμM—y%); ^c^—minimal inhibitory quantity (MIC); ^d^—IC_50_ against bovine acetylcholinesterase (bAChE); ^e^—IC_50_ against *Electrophorus electricus* acetylcholinesterase (eeAChE); ^f^—IC_50_ against human erythrocyte acetylcholinesterase (hAChE); ^g^—IC_50_ against mice hippocampus acetylcholinesterase; ^h^—against *Nilaparvata lugens* acetylcholinesterase; ^i^—IC_50_ against rat cortical acetylcholinesterase; ^j^—against *Torpedo californica* acetylcholinesterase; ^k^—against acetylcholinesterase not specified in the publication; ^l^—IC_50_ against human butyrylcholinesterase; ^m^—IC_50_ against *Equus caballus* butyrylcholinesterase; ^n^—against butyrylcholinesterase not specified in the publication. ALA—allanzanthane A; CAR—carvacrol; GAL—galanthamine; TAC—tacrine; HUP—huperzine A; BER—berberine; BERCl—berberine chloride; PHY—physostigmine (eserine); DEH—dehydroevodiamine; CHL—chlorpyrifos; DON—donepezil; NEO—neostigmine bromide; MCE—modified colorimetric Ellman’s method; CE—colorimetric Ellman’s method; BTLC—bioautography TLC; MCEF—modified colorimetric Ellman’s method and fluorescence measurement.

## 3. Activity

A comparison of the activity of individual isolated compounds is presented in Table 1.

Based on the information provided in Table 1, higher activity against AChE relative to galanthamine (**1**) is exhibited by the alkaloids aconorine, berberine (**7**), coptisine (**9**), 1,2-dihydrogalanthamine, epiberberine, jadwarine-A, jatrorrhizine, *N*-allyl-*nor*-galanthamine (**4**), *N*-(14-methylallyl)-*nor*-galanthamine (**5**), sanguinine (**6**), phthalates (e.g., bis (7-acetoxy-2-ethyl-5-methylheptyl) phthalate) and sterols (haloxylon B); relative to berberine (**7**), sargachromanol I (chromones) shows stronger inhibitory activity; relative to dehydroevodiamine, tiliroside and quercetin (flavonoids) have stronger inhibitory activity; compared to huperzine A, (7*S*,8*S*)-threo-4,9,9′-trihydroxy-8-O-4′-neolignan-7-O-*β*-*D*-glucopyranoside (lignans) has stronger inhibitory activity; compared to physostigmine (eserine), discorhabdin G (alkaloids) has stronger inhibitory activity; relative to neostigmine bromide, 7,8-didehydroorientalidine TFA salt and orientalidine (alkaloids) have stronger inhibitory activity; and compared to tacrine, 7-*epi*-javaniside, six diarylheptanoids from *Alpinia officinarum* and amentoflavone (flavonoids) show stronger inhibitory activity.

In the case of BuChE inhibitors, stronger BuChE inhibitory activity relative to galanthamine (**1**) is shown not only by the alkaloids aconorine, angustidine (**2**), angustine, angustoline, deoxyvobtusine, harmane, hohenackerine, jadwarine-A, nauclefine and pyrroloquinolone A, but also the bibenzyls bulbocol and gymconopin D; the coumarins bergapten, imperatorin (**17**), heraclenol-2′-O-angelate (**18**) and xanthotoxin; the phthalate bis (7-acetoxy-2-ethyl-5-methylheptyl) phthalate; the polyphenols vitisin A and heyneanol A; twelve phenanthrenes from *Bletilla strata*; the sesquiterpene lactones amberin, amberbin A and amberbin B; and the sterols haloxylon A and haloxylon B. In comparison to berberine (**7**), stronger inhibitory activity is exerted by sargachromanol I (chromones), 3*β*,21*β*,29-trihydroxyserrat-14-en-3*β*-yl p-dihydrocoumarate (triterpenoids) and compound 974-B (phlorotannins); relative to physostigmine, discorhabdin C and G (alkaloids) and diplacone (**30**) (flavonoids) show stronger inhibitory activity; relative to neostigmine bromide, alborine, isothebaine and *N*-methylisothebainium (alkaloids) have stronger inhibitory activity; and relative to tacrine, 7-*epi*-javaniside (alkaloids) has stronger inhibitory activity.

There are compounds that act more selectively and more potently on AChE versus (vs.) BuChE. The majority of them are alkaloids, including alborine, 9-O-demetil-2-α-hydroxyhomolycorine, 7,8-didehydromecambridine TFA salt, 7,8-didehydroorientalidine TFA salt, dihydroberberine (**8**), discorhabdin B, G and L, chlidanthine, hendersine B, hydrohydrastinine, 10-hydroxy-infractopicrin, 11-hydroxygalanthine, infractopicrin, mucroniferanines H, narcissidine, orientalidine, sanguinine (**6**), sargachromanol G and I, and vincosamide from *Buxus hyrcana* (except 31-demethylcyclobuxoviridine and papillozine C). Additionally, ethyl asterrate, methyl asterrate (anthranoids), (−) alpininoid B (**23**), (4*E*)-1,7-diphenyl-4-hepten-3-one (**24**) and other diarylheptanoids from *Alpinia officinarum* (diarylheptanoids), sargachromanol G and I (chromones), (2*E*,4*E*,6*R*)-6-hydroxydeca-2,4-dienoic acid (fatty acid), quercetin-3-*O*-neohesperidoside (flavonoids), methyl lucidenate E2, n-butyl lucidenate A and, n-butyl ganoderate H, ganoderic acid E, garcineflavanone A, lucidanol, lucidenic acid, lucidumol B (lanostane triterpenes), macelignan (lignans), creoside IV (polyphenols), amberbin C (sesquiterpene lactones), 3*β*,21*α*-diacetoxyserratan-14*β*-ol and 21*β*-hydroxyserrat-14-en-3,16-dione (triterpenoids) represent the same feature.

The following compounds are more selective and act more potently on BuChE vs. AChE: angustine, angustidine (**2**), angustoline, 31-demethylcyclobuxoviridine, harmane, isothebaine, lupanine, 2-methoxyatherosperminine, 4-methoxy-1-methyl-2-quinolone, narcipavline, nauclefine, pancratinine-C, papillozine C, pyrroloquinolone A, strictosamide (alkaloids), acetylvismione, bianthrone 1a, 2-geranylemodin, 3-geranyloxyemodin anthrone, 3-prenyloxyemodin, 3-prenyloxyemodin anthrone (anthranoids), bibenzyls from *Bletilla striata* (bibenzyls), bergapten, imperatorin (**17**), heraclenol-2′-O-angelate (**18**), xanthotoxin (coumarins), diplacone (**30**), 6-geranyl-3,3′,5,5′,7-pentahydroxy-4′methoxyflavane, 6-geranyl-3′,5,5′,7-tetrahydroxy-4′methoxyflavanone, 3′-O-methyldiplacol, 3′-O-methyldiplacone, 4′-O-methyldiplacol, 4′-O-methyldiplacone (**33**), mimulone (flavonoids), heyneanol A, vitisin A (polyphenols), cremaphenanthrene F and phenanthrenes from *Bletilla striata* (**37**,**38**) (phenanthrenes), amberin, amberbin A, (sesquiterpene lactones), leucisterol (steroids) and 3*β*,21*β*,29-trihydroxyserrat-14-en-3*β*-yl p-dihydrocoumarate (triterpenoids). On the basis of the IC_50_ value (µM) for galanthamine (**1**) obtained in the study and presented in Table 1, the median for AChE was determined, and it was IC_50_ = 0.94 µM, and for BuChE, it was 8.70 µM. It was assumed that galanthamine (**1**) exhibits strong inhibition of AChE and BuChE, and the potency of other inhibitors was compared to the value of the determined median. Those with IC_50_ under 1.5 μM were considered strong, those under 20 µM were medium-strong, and those between 20 and 100 µM were weak for AChE. For BuChE, those with IC_50_ under 10 μM were considered strong, those between 10 and 50 μM were medium-strong, and those in the range of 50–150 μM were weak.

From the presented tabular comparison (Table 1) of the results of the conducted studies (values of IC_50_), it follows that the compounds belonging to the alkaloid group exhibit the strongest activity against AChE and therefore are discussed in more detail. Fourteen of them have strong inhibitory activity with an IC_50_ value < 1.5 µM, and forty-two have medium-strong activity below 20 µM (Table 1).

The best AChE inhibition result in the entire table (Table 1) was obtained for sanguinine (IC_50_ = 0.007 µM). This was confirmed in another independent study in which the compound was derived from a different plant material (IC_50_ = 0.10 µM). Strong activity against AChE was also detected for five other Amaryllidaceae alkaloids (IC_50_ = 0.16 µM, 0.18 µM, 0.19 µM, 0.67 µM, 0.99 µM).

The different values of the inhibition coefficient obtained for sanguinine (**6**) are probably due to the use of various origins of AChE in the two studies. Similarly, for the same Amaryllidaceae alkaloids, e.g., lycoramine, inconsistent results were observed, with potent activity against *Electric eel* AChE and inactivity or weak activity using human AChE (isolated from *Narcissus jonquilla* ‘Pipit’ and *Narcissus poeticus* ‘Pink Parasol’).

Stronger and more potent AChE inhibitory activity than galanthamine (**1**) was obtained for five other isoquinoline alkaloids of the protoberberine type (IC_50_ = 0.52 µM, 0.53 µM, 0.51 µM, 0.74 µM, 0.80 µM) isolated from *Mahonia bealei* and *Mahonia fortunei*, as well as medium-strong activity for three *Mahonia* alkaloids (IC_50_ = 5.07–13.3 µM). 

Values of the IC_50_ inhibition coefficient of AChE below 1.5 μM were demonstrated by alkaloids derived from the sponge *Latrunculia biformis* (discorhabdin G) and an alkaloid from *Lycopodium*, huperzine C, with a slightly weaker result than the known activity of huperzine A.

Fourteen alkaloids from Ranunculaceae exhibit strong or medium-strong AChE inhibition values (IC_50_ = 2.51–12.1 µM), including an isoquinoline alkaloid, dihydroberberine (**8**), with IC_50_ = 1.18 µM (from *Coptis chinensis*), and diterpenoid alkaloids, including aconorine (from *Aconitum laeve*) and jadwarine-A (from *Delphinium denudatum*), with a potential stronger than the reference galanthamine (**1**) (Table 1). 

Similar moieties that are crucial for the binding of the inhibitor to the enzyme are also present in other strong and medium-strong inhibitors from other groups of compounds present in Table 1.

Lipophilic substituents will have a stronger affinity for the hydrophobic AChE ester part; hence, they show stronger binding to the enzyme and greater inhibition, which is described in more detail in the chemistry–structure–activity section [1,43].

A BuChE inhibition study showed inhibitory activity for thirty-one alkaloids with inhibition coefficients ranging from 10 to 50 µM and strong activity for alkaloids with inhibition coefficients below 10 µM. Alkaloids isolated from *Nauclea officinalis* (IC_50_ = 1.03 µM, 4.98 µM, 7.70 µM), including angustidine (**2**), have the lowest inhibition coefficients of all the alkaloids listed in Table 1.

The group of alkaloids is distinguished by a strong inhibitory effect on BuChE: alkaloids isolated from *Papaver somniferum* (IC_50_ = 2.80 µM, 7.1 µM), including one about thirty times more potent than neostigmine; two alkaloids stronger than galanthamine (1) from *Aconitum laeve* (IC_50_ = 8.72 µM, 9.94 µM) and deoxyvobtusine (IC_50_ = 6.20 µM) from *Voacanga globosa*; two alkaloids more potent than physostigmine, i.e., discorhabdin G (IC_50_ = 7.00 µM) from *Latrunculia bocagei* and 2-methoxyatherosperminine from *Cryptocarya griffithiana* (IC_50_ = 3.95 µM); and two alkaloids more potent than tacrine from *Uncaria rynchophylla* (Table 1).

From the coumarin group, coumarins isolated from *Mesua elegans* (IC_50_ = 0.70 µM, 1.06 µM, 3.06 µM) have the strongest ability to inhibit AChE. Four of them exhibit medium activity with an IC_50_ value <10 µM. Their activity against BuChE has not been studied. However, other presented coumarins displayed in the results seem to show BuChE inhibition (bergapten, imperatorin (**17**) and xanthotoxin (Table 1)). The IC_50_ values of coumarins isolated from *Archangelicae officinalis* and *Citrus hystrix* (Table 1) prove their activity against BuChE (IC_50_ from 7.5 to 23 µM) as well.

From all of the presented flavonoids, linarin from *Buddleja davidii* requires the minimal inhibitory quantity (10 ng) to inhibit AChE. Diplacone (**30**) and quercetin-3-O-neohesperidoside demonstrate the strongest activity against AChE, as determined by their IC_50_ values (IC_50_ = 7.2 µM, 6.98 µM). Medium-strong inhibitor values are reported for quercetin and tiliroside from *Agrimonia pilosa* and five flavonoids from *Prunus padus* var. *seoulensis* (IC_50_ between 15.49 and 21.53 µM) (Table 1). Flavonoids isolated from *Paulownia tomentosa* show relatively medium or weak activity against AChE (values of IC_50_ between 7.2 µM and 109.2 µM) and significant activity against BuChE (the strongest compounds demonstrated IC_50_ =1.4 µM, 3.8 µM). Garcineflavonol A (IC_50_ = 14.50 µM) showed medium-strong activity against BuChE. Lanostane triterpenes from *Ganoderma lucidum* showed medium activity against AChE, ranging from 9.40 µM to 31.03 µM, and n-butyl ganoderate H reached a value of IC_50_ = 9.40 µM. However, most of the results against BuChE are IC_50_ > 200 µM, which may indicate the selective activity of these compounds on AChE. Conversely, cremaphenanthrene F (phenanthrenes) from *Cremastra appendiculata* shows more potent inhibition against BuChE vs. AChE. Two lignans from *Camelia sinensis* var. *sinensis* ‘Huangjinya’ revealed strong AChE inhibition, which was higher or slightly weaker than huperzine (Table 1). Strong activity against BuChE and medium-strong activity against AChE were achieved for another lignan: macelignan from *Myristica fragrans*. The phlorotannin compound 974-B reached satisfactory results for both cholinesterases (for AChE IC_50_ = 1.95 µM and for BuChE IC_50_ = 3.26 µM).

Similarly, phthalates from *Lonicera quinquelocularis* had IC_50_ = 1.65 µM and 3.43 µM for AChE and IC_50_ < 10 µM for BuChE. Among diterpenes, dihydrotanshinon I and cryptotanshinone (**40**) showed strong inhibition against AChE, and triptexanthoside C (**45**) (xanthones) showed significant inhibition. Xanthonoids from *Garcinia mangostana* had IC_50_ on AChE from 2.5 μM, with six compounds having IC_50_ < 20 μM, and IC_50_ on BuChE from 0.7 μM, with six compounds having IC_50_ < 12 μM. Anthranoids from *Psorospermum glaberrimum* demonstrated medium-strong activity toward BuChE (9.25–13.30 μM) and weak activity toward AChE. A fatty acid from *Lycopodiella cernua* has shown high inhibition of AChE (0.22 μM). Remarkable results are also shown by polyphenols from *Camellia sinensis* var. *assamica* (caffeoylated catechin) against AChE, as well as by polyphenols from *Vitis amurensis* (heyneanol A, vitsin A), which had strong activity against AChE and BuChE. Medium-strong inhibition of AChE by creoside IV from *Codonopsis pilosula* and strong inhibition against BuChE by broussonin A (*Anemarrhena asphodeloides*) were found. High inhibition values against AChE and BuChE were also observed for sesquiterpene lactones from *Amberboa ramosa* (amberin, amberbin A and amberbin B). Sterols (leucisterol, haloxylon A and haloxylon B) from *Haloxylon recurvum* have shown strong inhibition of both BuChE and AChE. A strong inhibition value against AChE and medium inhibition against BuChE were shown by chromones from *Sargassum siliquastrum* and one of the diarylheptanoids: (−)-alpininoid B (**23**) (Table 1). Terpenoids demonstrated strong (*Lycopodiella cernua*) or medium-strong (*Lycopodiella cernua* or *Garcinia hombroniana*) activity against AChE and BuChE. All results are presented in Table 1.

## 4. Analysis Methods

The studied compounds occur in materials of natural origin in the form of mixtures. To determine the change in enzyme activity due to a particular compound, it is necessary to purify samples or even fractionate them. Studies of inhibitory activity toward cholinesterases in scientific reports are performed according to different methods and procedures. Nevertheless, in most cases, analyses are based on Ellman’s assay [48]. A summary of analysis methods used in the selected studies of cholinesterase inhibition is presented in Table 1. The description of the most important one is presented below.

The method should be simple, quick to perform, sensitive and inexpensive [77,137]. The analysis methods are based on a colorimetric assay using chromatographic techniques, TLC and fluorimetric and spectrophotometric measurements.

These methods are based on measuring changes in parameters indicative of enzyme activity before and after the introduction of the inhibitor to the system. Even slight changes in temperature, incubation time, pH, the concentration of substrates and the enzyme and the presence of other interfering compounds (detergents and heavy metal ions) can affect the accuracy of the results.

### 4.1. The Colorimetric Method of Ellman (1961)

This procedure is based on the result of the color reaction between the formed pre-thiocholine and the DTNB color developer (5,5′-dithiobis-(2-nitrobenzoic acid). Thiocholine is the product of the enzymatic reaction between acetylthiocholine (ATCI) and ChE. The intensity of the color of the product measured colorimetrically allows the determination of changes in enzyme activity. In the presence of an inhibitor, the change is suppressed, and we observe a lower-intensity color or complete inhibition [48].

Ellman’s method, among others, was applied to study the inhibitory activity of hexane extracts of the roots of *Archangelicae officinalis* L. against AChE and BuChE using physostigmine as a standard and the following conditions: AChE (0.45 U mL^−1^) in Tris-HCl buffer (pH 7.8); incubation of the enzyme at 4 °C for t = 30 min; and incubation of the reaction mixture at 37 °C for 20 min, followed by measurements using an ELISA microplate reader (λ = 412 nm). A weak result of inhibition was achieved for AChE (Angelica root hexane extract (IC_50_ AChE = 315 ± 20 (µg mL^−1^) and fruit hexane extract (IC_50_ AChE = 73 ± 7 (µg mL^−1^)), but much higher inhibition was observed with regard to the BuChE root extract (IC_50_ BuChE = 16 ± 5 (µg mL^−1^)) and fruit hexane extract (IC_50_ BuChE = 9 ± 2 (µg mL^−1^)) [28].

### 4.2. Spectrophotometric Modification of Ellman’s Method

Ding et al. (2013) described a modification used to determine the inhibitory activity of flavonoids and ginkgolides B and C from the leaves of *Ginkgo biloba* against AChE and BuChE [111]. Only flavonoids inhibit AChE (results in Table 1). In the method of Park and Choi (1991), the supernatant from the brown planthopper maggot was prepared (which contains ChE) [110]; the homogenized supernatant (T = 4 °C, t = 30 min.) was prepared in phosphate buffer (pH = 7.0) and 0.1 % Triton X-100. Acetone solutions of the analyzed compounds and standard (chlorpyrifos) were mixed with the previously prepared solution containing the supernatant and analyzed in a 96-well microtiter plate after 1h. DTNB and ATCI were added. Then, the measurement of absorbance was performed (λ = 405 nm microplate reader). The activity is relative to the control reaction, assumed as 100 %, and to the test compounds replaced by the buffer. On the basis of the results, the IC_50_ was determined [110].

The spectrophotometric modification of Ellman’s method described by Senol et al. (2010) was used to verify the inhibition of the methanol extract and isolated compounds (imperatorin (**17**), xanthotoxin and bergapten) from the fruits of *Angelica officinalis* L. [99]. The inhibition of both cholinesterases was tested using an ELISA microplate reader; galanthamine (**1**) as a standard; AChE from electric eel; and BuChE from horse serum. The potent inhibition of BuChE was observed for both the extract (100 µg/mL—85.65 ± 1.49%) and each of the compounds (Table 1) [100]. Many of the compounds were tested by using various modifications of the spectrophotometric method; they differed in the incubation time, the equipment used, the concentration of reactants and the wavelength measurement. The inhibitors belong to different groups of compounds (Table 1).

Cholinesterase inhibitory activity was also identified by using a TLC technique. By comparing the methods performed using the microplate and TLC, as described in Rhee et al. (2001), it can be assumed that TLC methods are more sensitive [16]. Due to the advantages of the TLC approach (simple, inexpensive and accurate measurement), this review focuses on methods using this technique.

### 4.3. TLC Modification of Ellman’s Method

The modification of Ellman’s method has been described by Rhee et al. (2001) [16,48]. As a result of the disruption of ATCI by AChE, choline is formed, which constitutes a colored compound (5-thio-2-nitrobenzoate anion) with DTNB. The color intensity of the product is measured spectrophotometrically. The bands of the tested extract are developed on the TLC plate, and the band pattern is sprayed with a mixture of DTNB and then ATCI in Tris-HCl buffer (Trizma hydrochloride with bovine serum, pH = 8); the AChE enzyme is then applied (3 U mL^−1^; from electric eel). This results in a yellow background due to a diazo compound (5 min) with white trails, which indicates inhibition by the extract. The disadvantage of the method is the possibility of false-positive effects [16].

The modified method of Rhee et al. (2001) was used, inter alia, to evaluate the obtained compound (mahanimbine) and petroleum ether extract (10 mg mL^−1^)) from *Murraya koenigii*. The plates were developed with a mobile phase (petroleum ether: CHCl_3_, 50: 50 (*v*/*v*)) and, after drying, were sprayed with DTNB/ATCI, followed by the implementation of the basic method. The enzyme activity was measured using a 96-well microplate reader [16,48,76]. The procedure described by Rhee et al. (2001) was also used to investigate the inhibitory activity against ChE by the extract and compounds (10-hydroxy-infractopicrin and infractopicrin) isolated from the toadstool *Cortinarius infractus*. For the measurement, the following compounds were used: AChE from bovine erythrocytes or equine serum BuChE and tacrine, physostigmine and galanthamine (**1**) as standards (>100 µM). The results were determined using a 96-well microplate reader [61].

### 4.4. TLC Bioautography by Marston

A properly made plate with applied spots of extracts was sprayed with a prepared mixture with the enzyme AChE or BuChE (T = 4 °C in Tris-hydrochloric acid, pH = 7.8, with bovine serum albumin as a stabilizer) and incubated (T = 37 °C, 20 min; increased humidity).

Then, in order to carry out the detection, a mixture containing, inter alia, Fast Blue Salt and alpha-naphthyl acetate prepared ex tempore was sprayed. After incubation (1–2 min.), a purple background due to the diazonium dye was obtained, while white spots indicated inhibition caused by the applied sample. The clear differences in the background color and band color indicate inhibition [101].

### 4.5. TLC Bioautography by Mroczek

A TLC plate with spots of the tested extracts (appropriately prepared) and the standard (galanthamine (**1**)) was developed with an adequate mobile phase (here, CHCl_3_/MeOH/25 % NH_4_OH 8:1:1 *v*/*v*/*v*) containing 2-naphthyl acetate. After developing and thoroughly drying (10 min), the plate was sprayed with the prepared mixture containing AChE (3 U mL^−1^) in TRIS buffer (pH 7.8) and incubated (increased humidity, T = 37 °C).

Then, it was sprayed with a solution of Fast Blue B salt. White spots demonstrating inhibition were clearly visible on the dark purple background due to the azo compound and appeared quickly (1 min), and they were very persistent (for 24 h). The advantage of this method is the decreased usage of the enzyme and the shortened time required for its incubation (10 min) compared to other methods. The method is highly sensitive and fast [77].

This validation method was performed by the author for the determination of the inhibition of Amaryllidaceae AChE isolated from extracts from *Narcissus jonquilla* ‘Pipit’ and *Narcissus jonquilla* ‘Havera’ and purified extracts of *N. jonquilla* ‘Baby Moon’, *Crinum moorei* and *Scadoxus puniceus*. This procedure manages to achieve high sensitivity. The inhibitory activity of the isolated alkaloid was demonstrated, and it was indicated that dihydrogalanthamine has greater inhibition, approximately 42% higher than galanthamine (**1**) [77]. With the application of this method, the activity of alkaloids present in the extract from *Argemone mexicana* L. roots was proved; it was weak for magnoflorine and strong for berberine (**7**), palmatine and galanthamine (**1**), isolated for the first time from the Papaveraceae family [138]. Additionally, a two-dimensional thin-layer chromatography/high-performance liquid chromatography/electrospray ionization time-of-flight mass spectrometry (TLC/HPLC/DAD/MS) system has been developed for both qualitative and quantitative analyses of active AChE inhibitors in plant samples [139]. The method of bioautography by Mroczek confirmed the inhibition of AChE by Amaryllidaceae alkaloids and determined their numerous occurrences in three cultivars of *Narcissus*: *N. jonquilla* ‘Baby Moon’, *N*. ‘Golden Ducat’ and *N*. ‘Cheerfulness’; the alkaloids were and identified both by using a TLC plate assay and by using TLC/HPLC/DAD/MS [140]. These methods have also been used to demonstrate AChE inhibitory activity and to qualitatively evaluate Lycopodiaceae alkaloids, and they were successfully used to study neuroprotective polyphenols from two species of *Trifolium* as well [141,142].

### 4.6. Fluorimetric Methods

These are fluorescent techniques (quenching) that measure enzyme–inhibitor binding affinities. This type of pathway has been chosen to demonstrate the activity of flavonoids from *Paulownia tomentosa* fruits with minor modifications to the spectrophotometric method of Ellman (1961). As a reference standard, physostigmine (eserine) was used (Table 1). In addition, using the fluorescence assay method (decrease), the affinity of the compounds with the relevant enzyme was studied.

The results were based on the dependency of the constant affinity rate, proportional to the inhibitory activity. Spectrophotometer measurements of the fluorescence emission were taken with a camera (M Series Multi-Mode Microplate Readers) (T = 18° and 37 °C) as the solution was titrated with a predetermined amount of a solution of hAChE (phosphate buffer (pH 8.0) (5 U mL^−1^)) with successive amounts of the tested flavonoids added. Studies have shown that the presence of a geranyl substituent at the C6 position in the structure of flavonoids is important for their ability to inhibit AChE [34]. 

The fluorimetric method was a part of the analysis of the *Mangosteen* seedcase extract outlined below [136]. To measure the compounds, the following steps were performed: the supernatant was centrifuged (12,000 rpm, 10 min.), a mixture with a buffer solution of ChE (5 μL) was added to the extract solution (20 μL), and the extract (CHCl_3_ in MeOH) was incubated (T = 37 °C, t = 30 min.). The supernatant (2 μL) was analyzed using ultra-performance liquid chromatography coupled with a photo-diode array detector and quadrupole time-of-flight mass spectrometry (UPLC/PDA/QTOF/MS), and the result was compared with that of the analysis without the enzyme. In the chromatogram, the peaks of mangostanol, allanxanthone E, gudraxanthone, γ-mangostin, 8-deoxygartanin and α-mangostin vanished (results in Table 1), so those compounds show an affinity for the enzyme. Then, the inhibitory activity of both cholinesterases was measured using a modification of Ellman’s method (Table 1). Using a fluorescence technique (quenching), affinity toward AChE was compared with γ-mangostin (Table 1) and 9-hydroxycalabaxanthone (IC_50_ > 100 µM). The first compound gained a much higher score. The authors supposed that the significant inhibition of AChE can respond to the presence of more than one prenyl group [136].

The methods presented in this review for determining cholinesterase inhibition by the investigated compounds can be described as qualitative and quantitative ones. Those based on the TLC technique (TLC bioautography) are more suitable for demonstrating inhibition by particular compounds (qualitative), and they are more sensitive compared to spectrophotometric methods (modifications of Ellman’s method). Nevertheless, they are not suitable for the determination of the inhibition coefficient, or it is difficult to measure. Therefore, they do not offer the possibility to compare the potency of inhibition among inhibitors. Both of these advantages are realized by methods based on a combination of the TLC technique (TLC bioautography) with more advanced techniques, such as HPLC/DAD/MS (high performance liquid chro-matography with photodiode array mass spectrometry), as mentioned in this article. Their use is increasingly observed in newer publications on cholinesterase inhibitors.

## 5. Conclusions

Reviewing the available publications, it can be concluded that methods for investigating cholinesterase inhibition have been mostly based on known procedures. These are generally modifications of previously used methods. They differ in parameters, which could affect the result of the activity of the enzyme and substrate, the incubation time, the method of analysis, the order of the addition of reactants and the type of assay.

It is important to study pure plant materials from respectively tested sources (heavy metal ions and detergents) and adequately purify the sample. The results (IC_50_) of the same compound when determined relative to hAChE and eeAChE can differ [136]. In some of the publications, the type of cholinesterase used in the study is not described or this information is difficult to obtain.

It is only possible to compare the IC_50_ values of specific compounds when determined under relatively similar conditions, using the same methods and compared to the same reference compound, thereby concluding potency. In most cases, one method is used, and results are rarely confirmed by using another method. An increased number of studies examining the inhibitory effects on both cholinesterases would be advantageous. It is beneficial to enhance the awareness and understanding of the subject of IChEs and activity measurement methods. Some of the studies did not include designations of activity toward both cholinesterases. It would be useful to use several reference substances in one study, which would allow for a better comparison of the available inhibition results.

However, more recent studies include the determination of the inhibition of both cholinesterases by the studied compounds and also attempt to analyze the structure and enzyme–inhibitor interaction, which is highly beneficial. This review reveals that inhibitors more potent than galanthamine (**1**), acting against both cholinesterases, are still being discovered. At the same time, compounds exhibiting potent selective activity against one of the cholinesterases have emerged. According to the established criteria in the study, strong activity against AChE was shown by 27 compounds, medium-strong was shown by 93 compounds, and weak activity was shown by 77 compounds, while against BuChE, strong, medium-strong and weak activity was shown by 43, 68 and 22 inhibitors, respectively. The largest group of compounds with a strong effect on both AChE and BuChE, as shown by the tabular comparison, were alkaloids. Compounds from this group demonstrated the most potent inhibition of AChE. Especially strong inhibition results against both cholinesterases were demonstrated for alkaloids from the Amaryllidaceae and Papaveraceae families. The most potent BuChE inhibition was demonstrated by compounds from various groups: alkaloids, coumarins, flavonoids, phenylpropanoids, polyphenols, phenanthrenes, phthalates, sterols and steroids, triterpenoids, xanthonoids and also lignans or phlorotannins. The presented review, as well as a summary of the results of the inhibitors’ structure analysis, may be beneficial in the determination and planning of further stages of research for the presented compounds. These data may also be helpful in the search and synthesis of new semi-synthetic or synthetic derivatives, as well as new biologically active substances. 

Work on finding compound derivatives with more specific, preferable features that we find in plant materials has yielded positive results. The ability to modify them allows for even better parameters of the drug, such as greater activity, a better match to the receptor, mitigated side effects, a longer duration of action or a favorable method of production. The integration of phytochemistry and cooperative disciplines of molecular modeling and chemical synthesis provides an opportunity to find effective drugs. The studies conducted continuously demonstrate that compounds of natural origin are still abundant and carry a lot of possible solutions.

The observed persistent deficiency of effective therapies for neurological diseases, including AD, requires researchers to further search for new therapeutic substances. The presented review, conducted for the period from 2008 to 2022 years, shows that the search for and analysis of natural cholinesterase inhibitors have not been exhausted yet. After summarizing in vitro studies, the conclusion emerges that the potential for the use of cholinesterase inhibitors in therapeutics has not been fully explored. Only some of them have been tested in vivo, and for several of them, clinical studies have been attempted. The results presented in this publication indicate that natural sources are a huge reservoir in the search for new therapeutic substances, including cholinesterase inhibitors.

## Data Availability

Not applicable.

## Abbreviations

ACh	Acetylcholine
AChE	Acetylcholinesterase
AD	Alzheimer’s disease
ATCI	Acetylthiocholine
BuChE	Butyrylcholinesterase
ChE	Cholinesterase
DTNB	5,5′-Dithiobis-(2-nitrobenzoic acid)
eeAChE	Electrophors electricus acetylcholinesterase
e.g., (lat. exempli gratia)	For example
hAChE	Human erythrocyte acetylcholinesterase
IBuChE	Inhibitor of butyrylcholinesterase
IC_50_	Inhibitory concentration for which enzyme activity is equal to half-maximal
IChE	Inhibitor of cholinesterases
SAR	Structure–activity relationship
TLC/HPLC/DAD/MS	Thin-layer chromatography/high-performance liquid chromatography/electrospray ionization time-of-flight mass spectrometry
Tris-HCl	Trizma hydrochloride with bovine serum
UPLC-PDA-QTOF-MS	Ultra-performance liquid chromatography coupled with photo-diode array detector and quadrupole time-of-flight mass spectrometry
vs.	Versus
